# Innovative adsorptive remediation of MoS₂-QDs from wastewater by decorated hierarchical mesoporous calcite@chitosan hydrogel@graphene oxide nanocomposite

**DOI:** 10.1186/s13065-026-01811-3

**Published:** 2026-05-07

**Authors:** Mohamed E. Mahmoud, Mohamed F. Amira, Enass A. I. Saleh, Hany Abdel-Aal

**Affiliations:** Faculty of Sciences, Chemistry Department, Alexandria University, Alexandria, Egypt

**Keywords:** MoS_2_-QDs removal, Nanocomposite, Hierarchical mesoporous calcite, Chitosan hydrogel, Graphene oxide, Adsorption investigations and assessments

## Abstract

Molybdenum disulfide quantum dots (MoS₂-QDs) are widely used in energy, catalysis, and biomedical applications. However, their uncontrolled release into the environment can pose ecotoxicological risks, highlighting the urgent need for effective removal and treatment strategies. Therefore, the present study is focused on the design and development of an innovative nanocomposite, consisting of hierarchical mesoporous calcite (HMC) embedded within a chitosan hydrogel (CH) and reinforced with graphene oxide (GO) nanosheets (HMC@CH@GO). This multifunctional system is introduced for the first time as an efficient composite for removal and recovery of MoS₂-QDs from contaminated aquatic environments. Comprehensive structural and morphological characterizations confirmed the successful formation of a mesoporous nanocomposite featuring an average pore size of 15.2 nm. Adsorption studies showed that optimal MoS₂-QD removal occurred at pH 4, with a maximum adsorption capacity at 21 mg/g using a dosage of 20 mg and 100 mg/L initial concentration. The adsorption equilibrium was rapidly reached within 30 min, while the kinetic data were best fitted to the pseudo-second-order model, showing a high correlation coefficient (R² = 0.999 at 50 mg/L). Isotherm modeling showed the best fit with both the Freundlich (R² = 0.992) and Langmuir (R² = 0.984) models. The combined isotherm and kinetic results confirm that the process is primarily driven by physisorption, complemented by a chemisorption component. Thermodynamic analysis verified endothermic and spontaneous reaction achieving ΔH° values as 11.43 kJ/mol (50 mg/L) and 13.12 kJ/mol (100 mg/L), besides the ΔG° values were turned into more negative with increasing temperature. In practical applications, the HMC@CH@GO nanocomposite achieved removal efficiencies up to 84.0% from tap water, 73.0% from seawater, and 64.0% from industrial wastewater. Also, HMC@CH@GO was identified to retain a reasonable performance after four regeneration cycles, exhibiting a sustainable and efficient adsorbent for MoS₂-QDs remediation from contaminated aquatic environments.

**Supplementary Information:**

The online version contains supplementary material available at 10.1186/s13065-026-01811-3.

## Introduction

Quantum dots (QDs), typically ranging from 2 to 10 nanometers, have attracted significant scientific attentions due to their unique and outstanding optical properties and physicochemical behavior. Their size-dependent emission, broad absorption spectra, and high photostability have enabled their integration into numerous technological, biomedical and environmental applications [1]. However, despite their benefits, the increasing production and disposal of QDs have imposed serious environmental and health concerns, particularly when these low-size materials were permitted to enter the ecosystems without proper treatment [2]. The unintentional release of nanomaterials during product disposal or through industrial effluents may lead to adverse ecological effects and pose a potential risk to human health [3]. Therefore, the increasing recognition of the ecological and environmental risks associated with QDs have spurred significant interest in developing effective removal and remediation strategies [4]. Studies have shown that QDs may exert cytotoxic effects by disrupting cell division resulting in DNA damage, besides all of which can ultimately cause cellular death [5].

Since their initial synthesis in 2014, molybdenum disulfide quantum dots (MoS₂-QDs) have attracted great attentions owing to their favorable biocompatibility, photostability, and unique photochemical characteristics [6]. Moreover, MoS₂-QD nanomaterials have been applied across a broad spectrum of fields, including electrocatalysis, solar energy conversion, energy storage, advanced electronics, chemical sensing, bioimaging, and photothermal therapy for cancer, among others [7]. Various synthesis approaches were explored to produce MoS₂-QDs with tailored physicochemical properties on the basis of numerous approaches. Top-down synthetic techniques are correlated to chemical electrochemical, solvothermal, thermal mechanical, emulsion, and other hybrid methods. In contrast, bottom-up synthesis typically involves the hydrothermal reaction of molybdenum and sulfur-containing precursors [8]. While MoS₂-QDs are often considered less toxic than other heavy metal-based quantum dots (e.g., CdSe and PbS), recent studies have shown that they can still exert dose-dependent toxic effects on living systems. MoS₂-QDs can reduce cell viability by forming reactive oxygen species (ROS) causing serious damages in various mammalian and microbial cells. It has been reported that exposure to high concentration levels of MoS₂-QDs has been linked to DNA strand breaks and chromosomal aberrations, indicating genotoxic potential [9]. In vitro and in vivo studies reported that MoS₂-QDs may trigger inflammatory pathways, contributing to tissue damage and altered immune responses [10]. Studies have reported that MoS₂-QDs are toxic to aquatic organisms such as zebrafish embryos, algae, and daphnia, causing developmental delays, malformations, and oxidative stress [11, 12]. The nanoscale size and chemical stability of MoS₂-QDs may allow them to persist in aquatic and terrestrial environments. As such, developing effective remediation strategies for MoS₂-QDs has become an urgent scientific priority. Among the various removal strategies of QDs from contaminated media, adsorption and biosorption techniques have been extensively utilized and characterized by their cost-effectiveness and operational simplicity. Various materials, including activated carbon, biochar, and clay minerals, have demonstrated high efficiency as adsorbents for QDs through electrostatic forces and van der Waals interactions [13]. In addition, biological sorbents, including microbial biomass and algae, have demonstrated significant potential for QDs removal, owing to numerous surface hydroxyl, carboxyl, and amino functional groups. Bioremediation methods utilizing specific bacteria and fungi have gained attention as environmentally benign strategies via mediating the enzymatic degradation and oxidation-reduction transformations, contributing to the detoxification and immobilization of QDs in both soil and water environments [14, 15]. Chemical precipitation is another effective remediation method, particularly for QDs containing toxic heavy metal species as cadmium or lead. This approach involves the addition of chemical agents like sulfides or hydroxides to convert dissolved metal species into insoluble precipitates, which can be subsequently removed from solution [16]. Photodegradation techniques, involving exposure of QDs to ultraviolet or visible light in the presence of photocatalysts, have also been explored for the oxidative breakdown of QDs [17]. While effective, this method may result in the generation of intermediate by-products with residual toxicity, necessitating post-treatment monitoring. Collectively, these remediation techniques represent a multifaceted approach to mitigating the environmental and health risks posed by QDs. Currently, adsorption using nanocomposite-based materials is regarded as one of the most effective and straightforward approaches for water treatment, offering several advantages over conventional methods. These include low energy consumption, high removal efficiency, environmental compatibility, and minimal ecological footprint [18]. The performance of adsorption processes involving nanocomposites is significantly affected by several operational factors, including pH, dosage, temperature, and time. Adsorption mechanisms are generally categorized into two types; physical adsorption (physisorption) and chemical adsorption (chemisorption) [19].

Graphene oxide (GO)-based nanocomposites have shown great potential for detoxification of contaminated water, especially removal of nanoscale pollutants such as heavy metal-based QDs, dyes, and other emerging contaminants [20]. Owing to its unique two-dimensional structure, high specific surface area, and rich content of oxygen-containing functionalities (viz., hydroxyl, epoxy, and carboxyl), GO provides multiple adsorption active sites for complexation with a great number of pollutants [21]. Furthermore, the surface chemistry of GO can be readily modified or combined with other functional materials, such as polymers, metal oxides, and biopolymers, to enhance selectivity, adsorption capacity, and mechanical stability under varying environmental conditions [22]. The developed GO-nanocomposites facilitate pollutant removal through several types of interactions, offering multifunctional pathways for water purification. Studies have demonstrated the efficiency of GO-based composites in adsorbing nanomaterials, thereby minimizing their mobility and ecological impact [23]. In addition, the regeneration and reusability of GO-composites further contribute to their potential in sustainable and cost-effective water treatment systems. As such, they represent a versatile platform for developing next-generation technologies aimed at addressing challenges in water pollution control at the nanoscale [24]. On the other hand, calcium carbonate (CaCO₃) is a widely available, non-toxic, and cost-effective material recognized for its environmental compatibility. Traditionally employed as an industrial filler in products such as paints, plastics, and paper, it has recently attracted attention for its potential in environmental remediation, including adsorptive removal of metal ions, organic dyes, and fluoride sequestration [25]. However, the adsorption efficiency of bulk calcite is restricted due to its relatively low surface area and limited active sites. To address this limitation, hierarchical mesoporous calcite (HMC), composed of nanoscale subunits, has been developed, offering an enhanced surface area, besides more active sites [26]. This advanced form of calcite has demonstrated effective performance in the adsorption and recovery of silver nanoparticles (AgNPs) from aqueous matrices [27].

To the best of our knowledge, removal and recovery of MoS₂-QDs pollutant from wastewater was not previously explored or reported. Therefore and based on the previously mentioned facts about the urgent need for developing innovative and effective nanocomposite for removal of MoS₂-QDs as an emerging pollutant from wastewater, the current research is primarily devoted to synthesize and characterize a novel HMC@CH@GO hybrid nanocomposite via binding combination of HMC with GO, and chitosan hydrogel. HMC@CH@GO was additionally aimed to synthesize as an eco-friendly, multifunctional nanocomposite with improved physicochemical performance for high adsorption performance. This was aimed via integration of the enhanced surface area and porosity of HMC with the excellent dispersibility and adsorption potential of GO, besides the biocompatibility and chelating nature of chitosan hydrogel as a biodegradable polymer. The structural, compositional, and morphological properties of as-synthesized HMC@CH@GO were extensively investigated by diverse instrumentations. Furthermore, the current search is devoted to explore the adsorption behavior MoS₂-QDs onto HMC@CH@GO nanocomposite under varying experimental conditions and parameter to verify the optimum fitting to the evaluated adsorption kinetics and isotherm patterns to assess the feasible mechanisms. Ultimately, this work is principally aimed to offer a sustainable and effective nanotechnology-based solution for water purification from emerging nanomaterial contaminants as MoS₂-QDs.

## Materials and experiments

### Chemicals, materials and instruments

All chemicals utilized in this work, as listed in Table 1S, were all AR and used without prior purification. The instrumental techniques employed for the characterization, structural analysis, morphological evaluation and compositional determination of the as-synthesized nanocomposite are summarized in Table 2S.

### Synthesis and experiments

#### Synthesis of MoS_2_-QDs

Bulk molybdenum disulfide (MoS₂) was initially synthesized following a modified version of a previously reported procedure [28]. An aqueous precursor solution was prepared by dissolving 6.18 g of ammonium molybdate (AM) in 100 mL of distilled water (DW), added to 1.92 g of citric acid (CA) to achieve a 1:2molar ratio. The solution was stirred at 90 °C for 30 min to ensure complete dissolution of the reactants. Subsequently, 47.7 mL of 10% (w/v) ammonium sulfide (AS) was added dropwise under continuous stirring until a dark brown suspension was formed, indicating the precipitation of MoS₂. This was centrifuged at 3000 rpm for 1 h, and the resulting material was thoroughly rinsed with DW and then dried at 50 °C. Next, MoS₂-QDs were then synthesized via a facile one-step process involving ultrasonication-assisted liquid exfoliation followed by hydrothermal treatment, as described [29]. In this method, a total of 1.0 g of the synthesized MoS₂ powder was dispersed in 150 mL NMP (N-methyl-2-pyrrolidone), subjected to continuous sonication for 3 hand placed in an autoclave and heated at 200 °C for 36 h. MoS₂-QDs formation was realized by the appearance of a yellow solution. The suspension was then centrifuged at 8000 rpm for 40 min, and the upper three-fourths of the supernatant, containing MoS₂-QDs, was collected. Two stock solutions of MoS_2_-QDs of different concentration (50 and 100 mg/L) were prepared and stored at 0–4 °C for further use.

#### Synthesis of graphene oxide (GO)

Graphene oxide (GO) was synthesized from ultrapure graphite fine powder using a modified Hummers–Hoffman method [30]. Briefly, 15.0 g of graphite powder and 7.5 g of sodium nitrate were added to 324 mL concentrated sulfuric acid and 36 mL concentrated phosphoric acid in a 1 L round-bottom flask and stirred for 10 min on an ice bath. Subsequently, 45.0 g of KMnO₄ was gradually introduced while keeping under 5 °C and stirring was continued for 2 h. The reaction temperature was then increased gradually to 98 °C and maintained for 1 h, during which DW was slowly added to approximately 1.2 L. Finally, 45 mL H₂O₂ was added for quenching. The resulting GO suspension was collected, washed and dried.

#### Synthesis of hierarchical mesoporous calcite@chitosan hydrogel (HMC@CH)

Hierarchical mesoporous calcite (HMC) embedded within a chitosan hydrogel matrix was synthesized in situ following a modified procedure based on a previously reported method [31]. Briefly, 0.5 mol/L calcium chloride (CaCl₂) was dissolved in mixed solvent of ethanol and DW (1:2 v/v) to prepare solution (A) Separately, 0.5 mol/L Na₂CO₃ was prepared using the same solvent composition and volume to obtain solution (B) In parallel, 1.5 g of chitosan was dissolved in 250 mL DW containing 5 mL of concentrated acetic acid under continuous stirring at 60 °C until complete dissolution, forming solution (C) Next, 100 mL solution C was gradually added to the stirred solution A. Subsequently, solution B was slowly introduced under continuous stirring and maintained for 24 h. Afterward, 7 mL of glutaraldehyde was added dropwise to initiate gelation, followed by more 10 min stirring. The resulting HMC@CH composite was filtered under vacuum, washed thoroughly with a 1:2 (v/v) ethanol–DW mixture, and left to dry at ambient temperature for four days.

#### Fabrication of HMC@CH@GO hybrid nanocomposite

The incorporation of GO into HMC@CH matrix was carried out by following a modified procedure adapted from a previously reported method [32], employing silica gel as a catalyst. Specifically, 2.0 g GO, 3.0 g HMC@CH, 10 mL ethyl acetate, and 1.5 g silica gel were introduced into a clean round-bottom flask. The mixture was irradiated in a microwave for 2 min per cycle, repeated ten times to ensure effective interaction and composite formation. After completion of the reaction, silica gel was removed using a suitable mesh sieve and the resulting nanocomposite (HMC@CH@GO) was then thoroughly washed and dried.

### Adsorption studies

Batch experiments were established to estimate the efficiency of the synthesized HMC@CH@GO nanocomposite in removing MoS₂-QDs from aqueous solutions. The concentration of MoS₂-QDs was quantified by UV–Vis spectroscopy at λ = 360 nm using an external calibration curve prepared from standard dispersions in the range of 10–100 mg/L. The stock suspension was initially quantified by atomic absorption spectrophotometry (AAS) through measurement of total molybdenum content to ensure accurate concentration determination. Working solutions (50 and 100 mg/L) were subsequently prepared by dilution of the validated stock solution. In each experiment, 20 mg of the dried nanocomposite was mixed with MoS₂-QDs solution (10 mL). The volume was adjusted to 15 mL using DW, and the mixture was agitated on a mechanical shaker at 300 rpm for 30 min at ambient temperature. After filtration, the residual MoS₂-QDs were quantified. Adsorption experiments were conducted by using MoS₂-QDs (50 and 100 mg/L) and the removal (%) and capacity (q) were investigated using Eqs. (1) and (2), respectively.

1 $$\:\%\:Removal=\frac{Co-C}{Co}\times\:100$$

2 $$\:q=\frac{\left(Co-C\right)\:V}{m}\:\:\:\:\:\:$$

Where, C_o_ and C are the starting and remaining MoS_2_-QDs concentrations (mg/L), V (L) is the volume of adsorption mixture, m (g) mass of nanocomposite and q (mg/g) is the adsorption capacity.

The influence of pH was assessed by adjusting the pH of MoS₂-QDs solution at 2.0–8.0 by 0.1 mol/L HClor NaOH. All tests were carried out by following the same batch procedure as previously described. Furthermore, the point of zero charge of HMC@CH@GO nanocomposite was determined following the method described [33]. In brief, 100 mg HMC@CH@GO was dispersed in 50 mL of 0.1 mol/L NaCl, and pH values were regulated between 2 and 12. The suspensions were shaken for 4 h, and the final pHs were recorded after 24 h to calculate the ΔpH and determine the PZC.

To investigate the impact of HMC@CH@GO dosage, a series of adsorption experiments were conducted using 10.0, 20.0, 30.0, 40.0, and 50.0 mg, while maintaining the pH at 4 and contact time at 30 min.

Evaluations of temperature and thermodynamic parameters were assessed by performing adsorption experiments at varying temperatures (20–70 °C) at pH 4 and 30-min contact time. These studies provided insight into feasibility and nature of the adsorption process.

The effect of contact time was examined by changing the contact time between 1 and 35 min under optimal pH conditions (pH 4). Samples were collected at predetermined intervals to evaluate the removal efficiency as a function of time.

The effect of initial MoS₂-QDs concentration was performed to investigate by varying the initial concentrations of MoS₂-QDs (20, 30, 40, 50, 100, and 200 mg/L). All experiments were performed under the optimized conditions of pH 4 and 30-min contact time.

The reusability of the HMC@CH@GO was evaluated over successive adsorption–desorption cycles. For each cycle, 200 mg nanocomposite was combined with 10 mL of 100 mg/L MoS₂-QDs solution, diluted to 15 mL with DW, and agitated for 30 min at pH 4. After filtration, the nanocomposite was regenerated by sequentially subjecting it to 50 mL of 0.1 mol/L NaOH for 30 min followed by washing with 10 mL of DW, then subjected to 50 mL of 0.1 mol/L HCl for more 30 min, and finally washed with 10 mL of DW before reuse.

To assess the practical applicability of the developed HMC@CH@GO nanocomposite, adsorption experiments were performed on contaminated real water samples with MoS₂-QDs. Three different collected water samples were tap water (drinking), seawater and industrial wastewater. Each 10 mL water sample was spiked with 10 mg/L of MoS₂-QDs and treated with 50 mg of the nanocomposite without pH adjustment at realistic environmental conditions. After shaking for 1 h, the samples were filtered, and the percentage removal of MoS₂ QDs was determined. The effluent from each sample was then collected and treated again with a fresh 50 mg portion of the adsorbent. The removal percentage was calculated after the second treatment cycle. A similar third treatment cycle was subsequently performed, and the final removal efficiency was determined.

## Results and discussion

### Synthesis and structural assessment of HMC@CH@GO nanocomposite

**Fig. 1:**
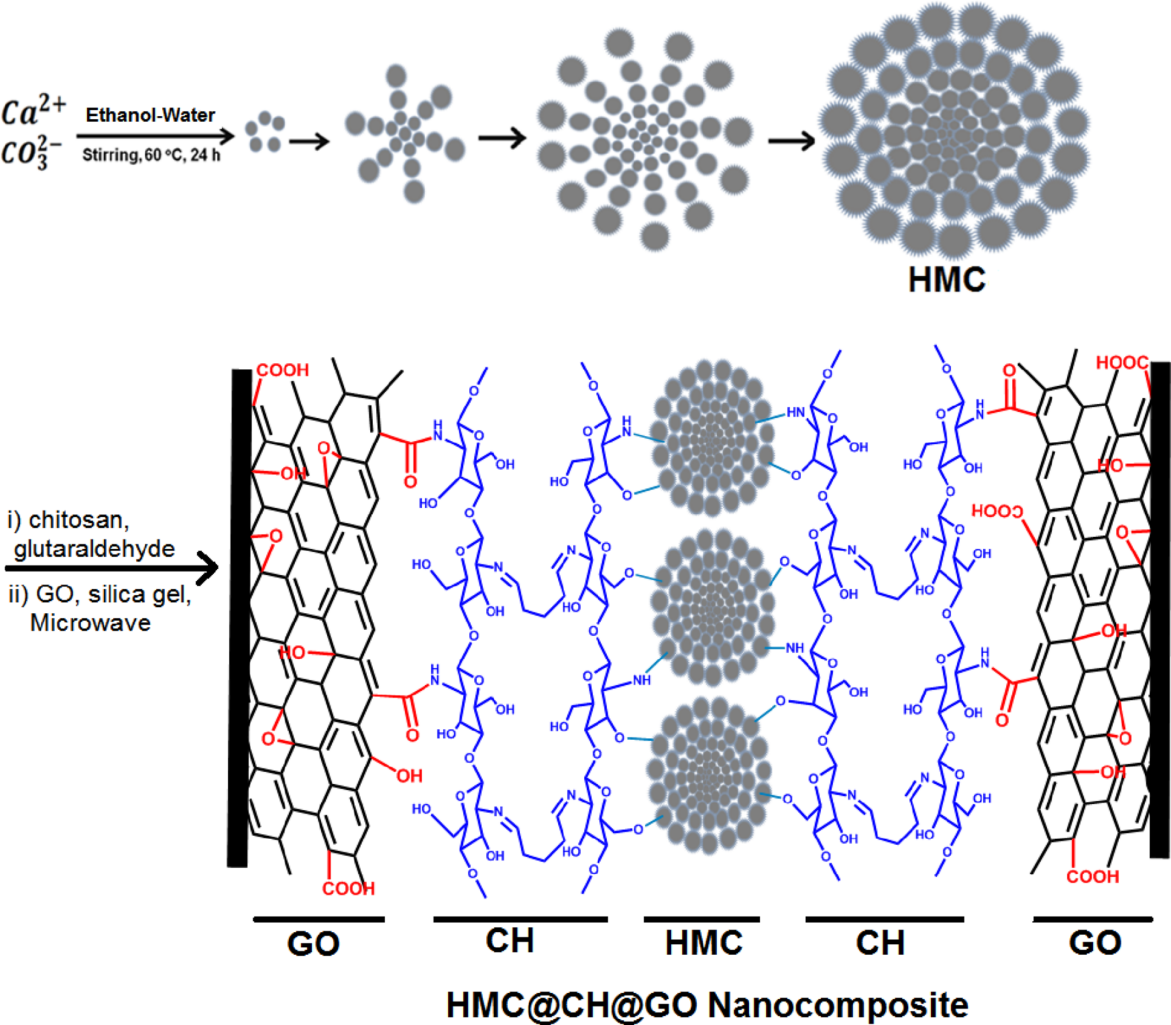
Schematic representation of synthetic procedure for preparation of HMC@CH@GO

The synthetic pathways for the hierarchical mesoporous calcite-embedded chitosan hydrogel–graphene oxide nanocomposite (HMC@CH@GO) involves a multi-step, integrated assembly strategy combining inorganic mesostructures, biopolymers, and carbon-based nanomaterials are illustrated in Fig. 1. The process begins with the synthesis of HMC via calcium (Ca²⁺) co-precipitation with carbonate (CO₃²⁻) ions in ethanol–water under controlled thermal stirring at 60 °C for 24 h. This route promotes nucleation and controlled growth of uniform spherical mesoporous calcite aggregates with internal pore networks ideal for hosting functional moieties. In a simultaneous step, chitosan hydrogel (CH), a rich biopolymer in amino and hydroxyl groups, is crosslinked using glutaraldehyde, facilitating the formation of a chemically stable hydrogel network. The crosslinking process also allows for the uniform dispersion of the HMC particles within the chitosan matrix, ensuring structural homogeneity and enhanced mechanical integrity. The glutaraldehyde acts by forming imine linkages (Schiff bases) between the aldehyde groups and primary amines of chitosan, resulting in a three-dimensional hydrogel framework. In the final stage of HMC@CH@GO nanocomposite synthesis, silica gel was used to play a pivotal role as a heterogeneous acid–base catalyst, facilitating the covalent integration between GO and HMC@CH network. GO is rich in oxygenated functional groups, particularly carboxyl (-COOH) and aldehyde (-CHO) groups, which can react with the primary amine (-NH₂) functionalities of chitosan through amide bond formation. The catalytic efficiency of silica gel is significantly enhanced under microwave irradiation providing rapid volumetric heating, reducing reaction time, and promoting molecular collisions for direct bond formation. Microwave energy accelerates the dehydration step of the amide condensation reaction, thus improving yield and covalent grafting efficiency. The combined action of silica gel (as a catalytic scaffold) and microwave-assisted heating ensures the formation of stable amide linkages (–CONH–) between GO and the chitosan hydrogel matrix, resulting in a robust and chemically integrated HMC@CH@GO hybrid network [34]. This covalent bonding enhances the mechanical stability, structural uniformity, and functional integration of the as-prepared nanocomposite for advanced material applications. This conception was further confirmed by the following structural analysis techniques.

#### FT-IR investigation

**Fig. 2:**
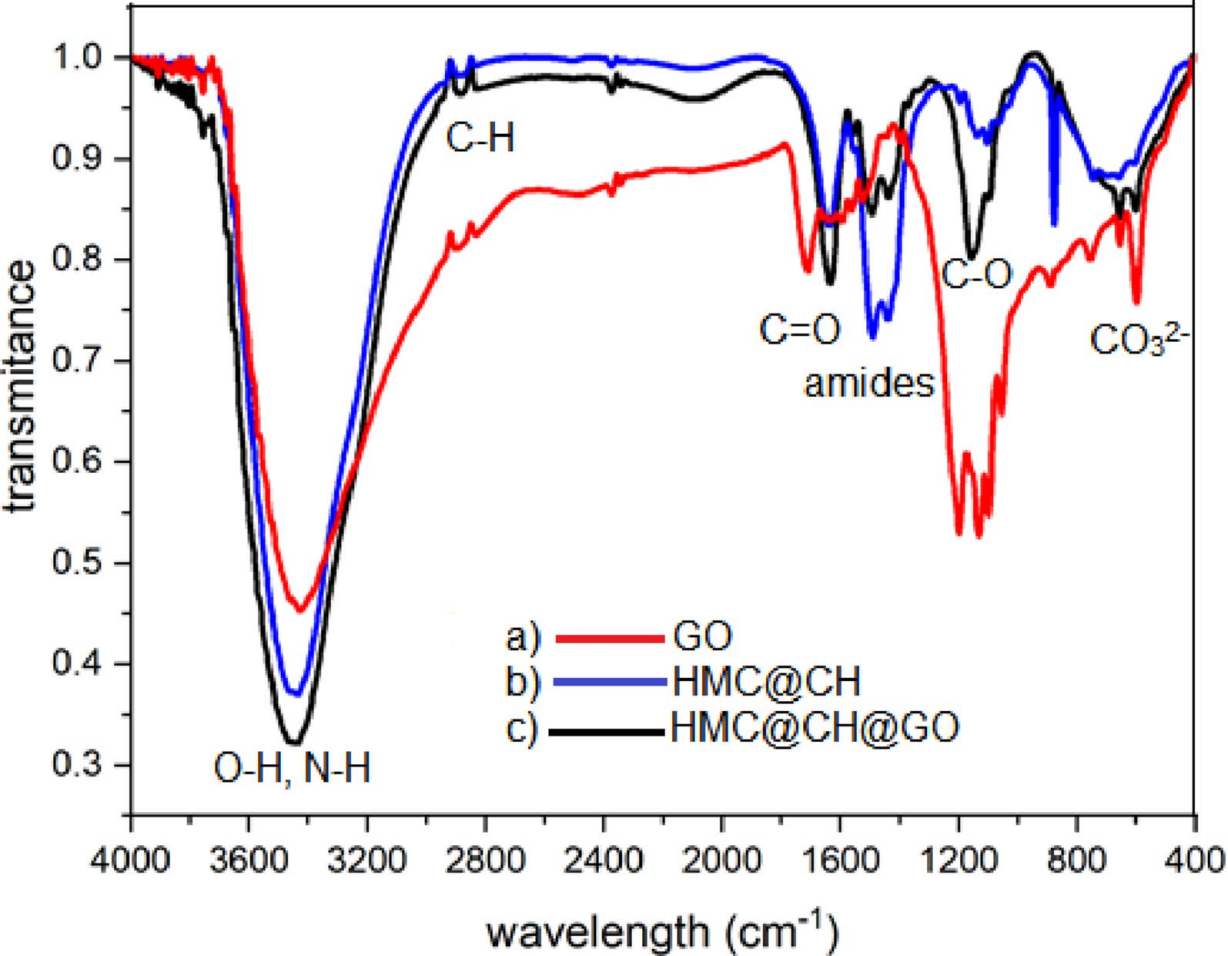
FT-IR spectra of (**a**) GO, (**b**) HMC@CH, and (**c**) HMC@CH@GO nanocomposite

The FT-IR spectral investigation was applied to elucidate the functional groups allocated on GO, HMC@CH, and HMC@CH@GO nanocomposite as represented in Fig. 2. The GO spectrum referred to characteristic absorption bands at 3426 cm⁻¹ (O–H stretching), 2897 and 2830 cm⁻¹ (C–H stretching), and a prominent peak at 1711 cm⁻¹ ascribed to C = O vibration of carboxyl groups. Additional peaks at 1631 cm⁻¹ (C = C stretching), 1521–1199 cm⁻¹ (epoxy and alkoxy C–O stretching), and below 900 cm⁻¹ confirmed the presence of oxygenated functionalities, validating the successful oxidation reaction of graphite into GO [35]. The HMC@CH spectrum, a broad band at 3435 cm⁻¹ indicated O–H and N–H vibrations, reflecting the presence of chitosan and H-bonding. The 2886 and 2833 cm⁻¹ peaks correspond to aliphatic chain C–H, while absorption bands at 1638 cm⁻¹ (amide I), 1491 and 1439 cm⁻¹ (amide II and III) confirmed the presence of primary amine groups [36]. The carbonate vibrational modes of calcite were evidenced by peaks at 877, 744, and 659 cm⁻¹. The FT-IR spectrum of the HMC@CH@GO revealed combinations of characteristic peaks from both GO and the Chitosan hydrogel matrix. The broad band at 3436 cm⁻¹ indicated the possibly enhanced hydrogen bonding. Shifts in the amide region (1634, 1558, and 1493 cm⁻¹) and the presence of new bands, such as at 2096 cm⁻¹, suggest strong interfacial interactions and successful integration of GO within the chitosan-calcite matrix [37]. The preservation of calcite vibrational bands and C–O related peaks supports the structural stability of the hybrid composite. These spectral modifications confirm the formation of a chemically interactive, multifunctional nanocomposite suitable for environmental remediation or advanced material applications.

#### XRD investigation

**Fig. 3:**
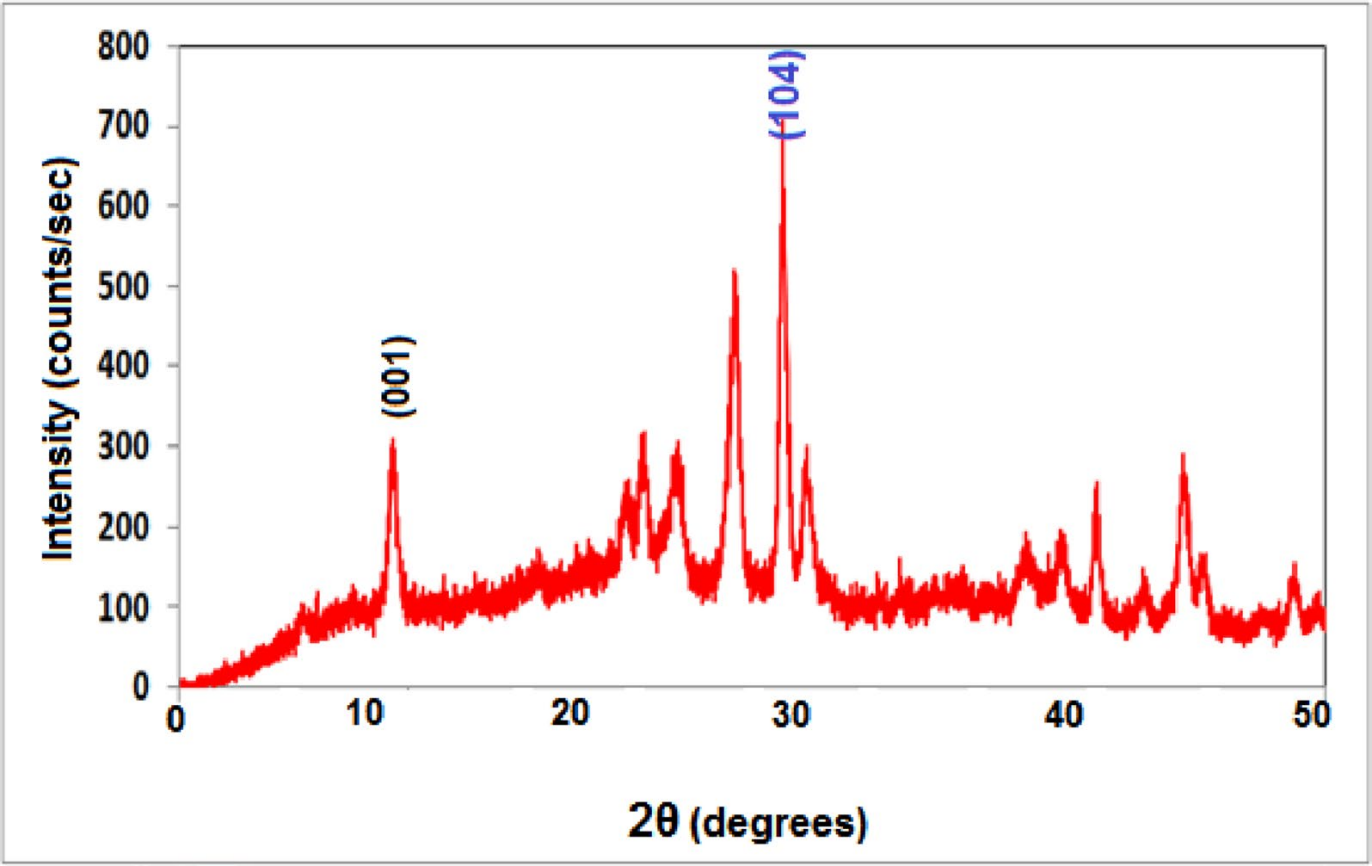
XRD pattern of HMC@CH@GO nanocomposite

The XRD pattern of s-synthesized HMC@CH@GO nanocomposite (Fig. 3), displays a series of distinct diffraction peaks within the 2θ range from 5° to 55°, indicating a semi-crystalline nature with contributions from both inorganic and carbonaceous phases. The most observed intense peak near 2θ = 29.5° corresponds to the (104) crystal plane of calcite (CaCO₃), owing to the presence of the rhombohedral crystalline phase of calcite. Additional reflections at approximately 2θ = 23.0° (012), 36.0° (110), 39.5° (113), 43.2° (202), 47.6° (018), and 48.5° (116) are also attributed to calcite, further supporting its incorporation within the hybrid nanocomposite [38]. The broad background hump in the lower angle region is indicative of the amorphous or semi-crystalline nature of chitosan and suggests the presence of disordered domains, which is typical for biopolymeric hydrogel matrices [39, 40]. Moreover, the lack of sharp diffraction peaks near 2θ = 10–12°, which typically corresponds to the (001) plane of graphene oxide (GO), suggests that GO nanosheets are well-exfoliated or highly dispersed within the hydrogel matrix. This suppression of the GO peak implies strong interfacial interactions with chitosan and calcite, possibly due to covalent bonding or hydrogen bonding, which disrupt the regular stacking of GO layers [41, 42]. The overall diffraction pattern reflects the successful integration of mesoporous calcite into the chitosan hydrogel network, while maintaining its crystalline identity. Simultaneously, the presence of GO in a dispersed or disordered form within the hybrid matrix enhances the nanocomposite structural complexity without significantly contributing to crystalline diffraction features. This combination of crystalline calcite, semi-crystalline biopolymer, and disordered GO underscores the hybrid nature of the material and supports its potential application in areas requiring structural stability, high surface area, and multifunctional performance.

#### Morphological and microstructural characterization

**Fig. 4:**
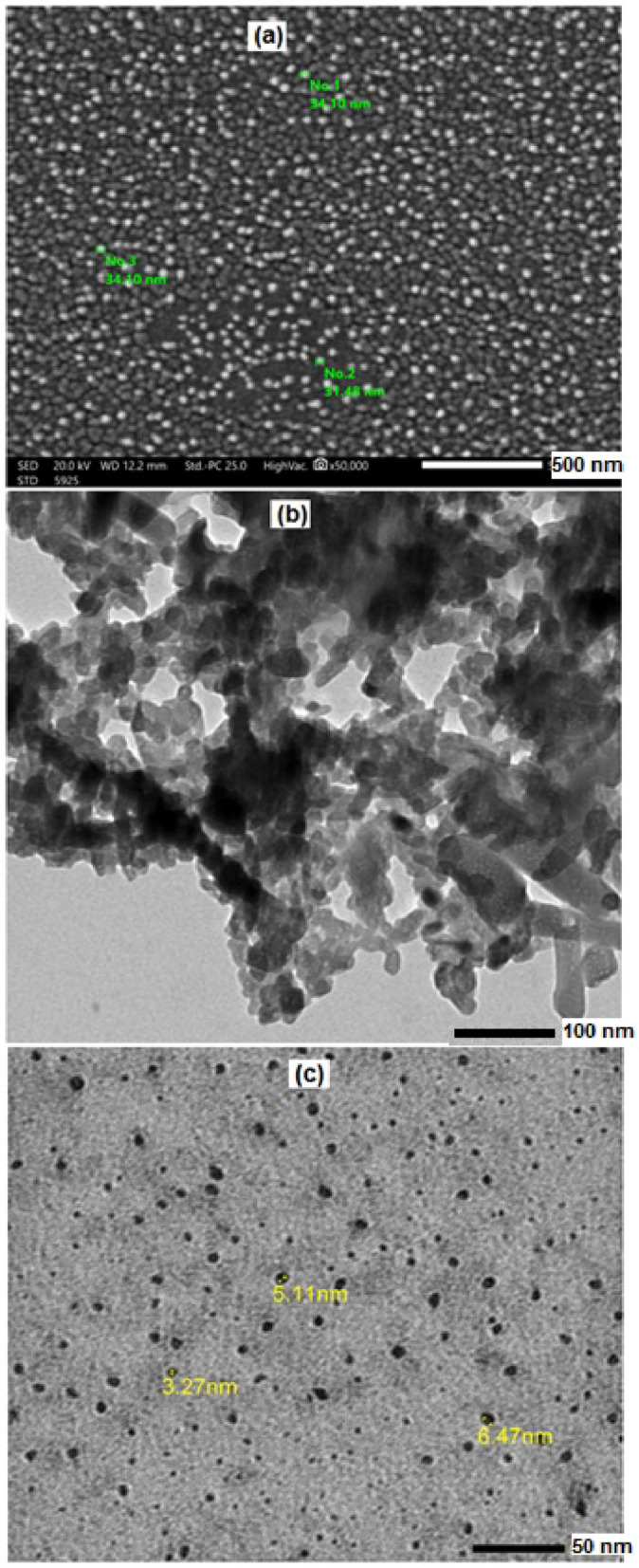
(**a**) SEM, (**b**) TEM of HMC@CH@GO nanocomposite, and (**c**) HR-TEM of MoS2-QDs

The surface morphology and microstructure of HMC@CH@GO were investigated using SEM and TEM, as shown in Fig. 4a and b, respectively. A high-resolution SEM image (×50,000 magnification) of the nanocomposite surface reveals a uniform distribution of spherical nanoparticles across the chitosan hydrogel matrix. The measured particle sizes, range from approximately 31.48 nm to 34.40 nm, indicating a relatively narrow size distribution. This homogeneity suggests successful incorporation of calcite nanoparticles into the hydrogel network without significant aggregation. The fine dispersion can be attributed to the stabilizing effect of the GO sheets, which likely facilitate uniform nucleation sites for calcite crystallization through their abundant oxygen-containing functional groups [20]. The TEM image further elucidates the internal nanostructure, highlighting the presence of highly dispersed nanoparticles embedded within a low-contrast, web-like matrix. The contrast variation and granular morphology suggest a hierarchical porous framework, characteristic of mesoporous calcite embedded within the chitosan-GO hybrid matrix. The dark regions correspond to the denser calcite nanoparticles, while the lighter, more diffuse areas indicate the surrounding organic chitosan-GO network [26]. Notably, the TEM image supports the SEM findings by confirming the nanoscale dispersion and connectivity within the nanocomposite. Furthermore, the observed porous architecture is beneficial for potential applications due to the enhanced surface area and accessible pore structure. Figure 4c displays HR-TEM image of the synthesized MoS₂-QDs. The image clearly reveals a large number of uniformly distributed, nearly spherical nanoparticles with well-defined boundaries. The measured diameters of selected MoS₂-QDs were at 3.27 nm, 5.11 nm, and 6.47 nm confirming that the synthesized structure fall within the quantum confinement regime, typically less than 10 nm. The high contrast and consistent shape suggest the formation of discrete MoS₂-QDs without significant agglomeration or clustering, indicating effective control over nucleation and growth during synthesis.

**Fig. 5:**
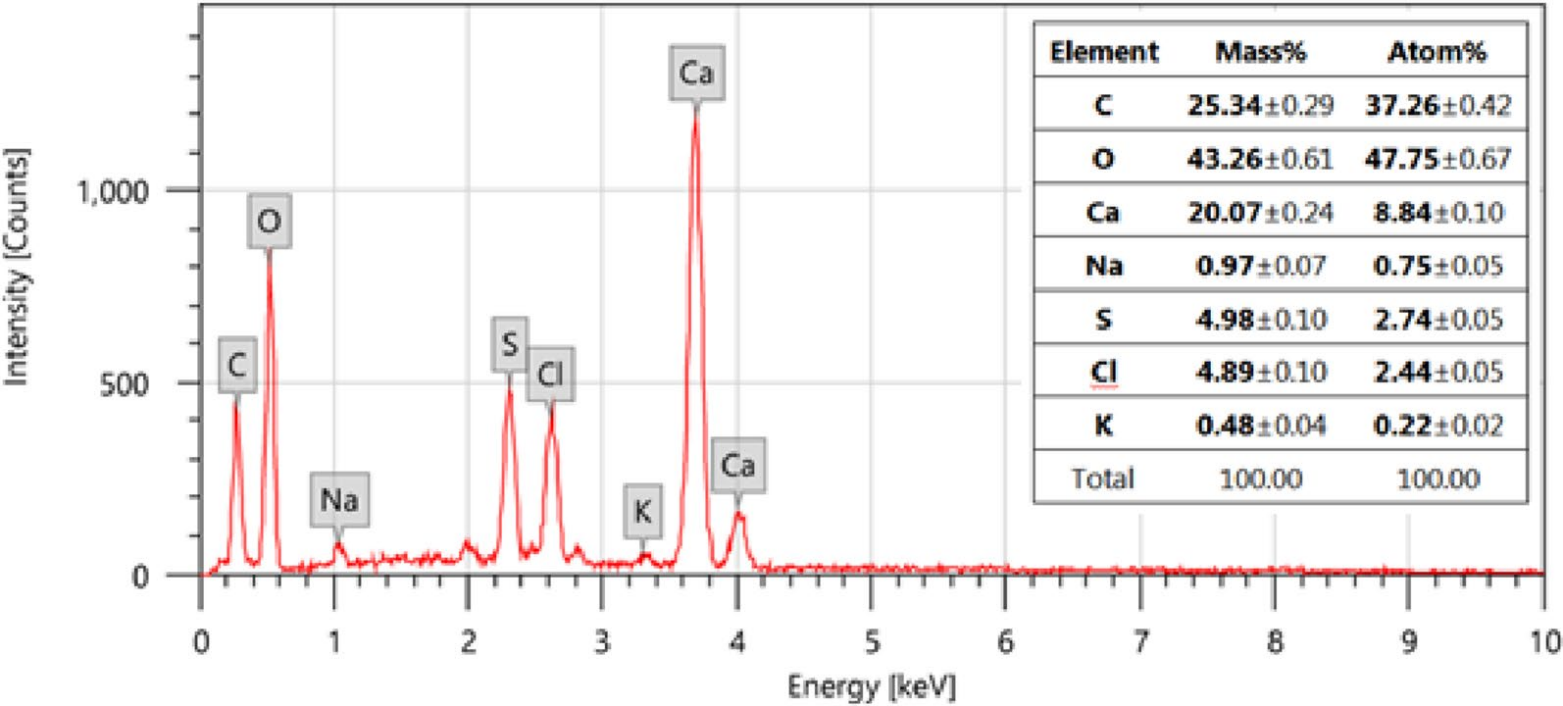
EDX analysis of HMC@CH@GO nanocomposite

**Fig. 6:**
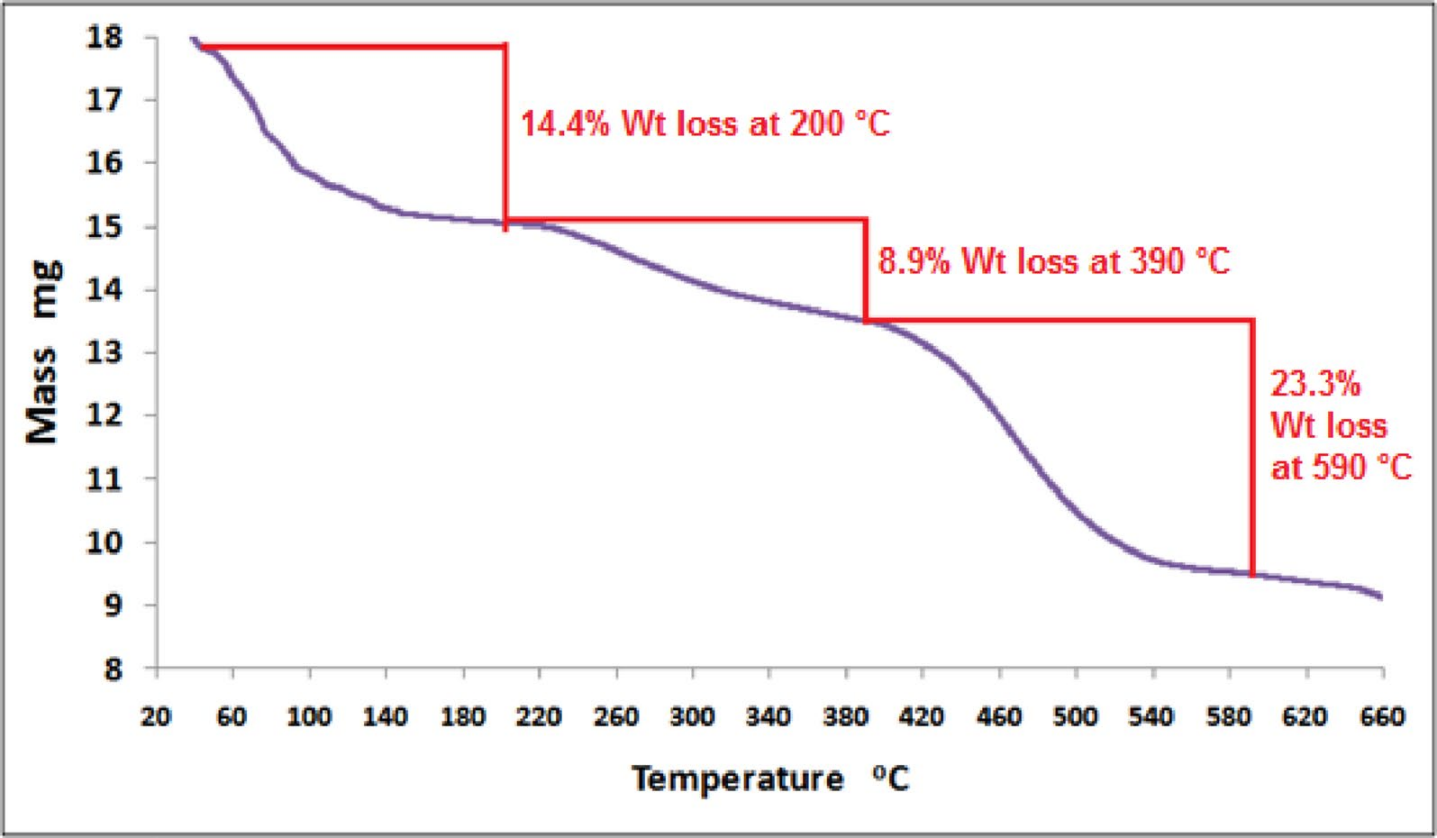
TGA of HMC@CH@GO nanocomposite

#### EDX analysis

The EDX spectrum of HMC@CH@GO (Fig. 5), confirms the presence of key elements consistent with the intended nanocomposite formulation, namely carbon (C), oxygen (O) and calcium (Ca). Carbon (25.34%) and oxygen (43.26%) were the dominant elements, confirming the successful integration of organic (chitosan and GO) and inorganic (calcite) components within the nanocomposite matrix. The substantial oxygen content reflects the abundance of oxygen-containing functionalities as hydroxyl, carboxyl, and epoxide moieties inherent in both chitosan and GO, as well as the carbonate groups of calcite. Calcium was detected at 20.07%, indicating the effective incorporation of mesoporous calcite into the hydrogel structure. The presence of sulfur (S 4.98%), chlorine (Cl 4.89%), sodium (Na 0.97%), and potassium (K 0.48%) signals, although present in minor intensities, may originate from reagents used during the GO synthesis and functionalization stages.

The elemental distribution derived from this EDX analysis, supported by SEM-TEM mapping, confirms the hybrid nature of the nanocomposite, combining the organic matrix with inorganic mineral phases, which is essential for enhancing the mechanical stability, adsorption performance, and chemical functionality of the composite material. The uniform dispersion of calcium and carbon further supports the successful embedding of mesoporous calcite within the chitosan-GO network.

#### Surface assessment

To evaluate the related specific surface area and pore characteristics to the as-synthesized HMC@CH@GO nanocomposite, nitrogen adsorption–desorption measurements were carried out at 77 K. The corresponding isotherm and BET (Brunauer–Emmett–Teller) plot are illustrated in Fig. 1Sa and 1Sb, respectively. The graphs exhibit a type IV isotherm accompanied by a distinct H3 hysteresis loop, indicating mesoporous structures as plate-like particles [43]. The steep increase in nitrogen uptake at high relative pressures (p/p₀ > 0.8) confirms the existence of mesopores, while the moderate adsorption at lower pressures reflects multilayer adsorption on the internal surfaces. The hysteresis loop also indicates capillary condensation, further confirming the mesoporous nature of the composite. The linear BET plot derived from the adsorption measurements in the pressure range from 0.05 to 0.25, indicate a moderate surface area (11.295 m²/g). It is mainly attributed to the porous architecture created by the incorporation of calcite nanoparticles and layered GO within the chitosan matrix. The computed total pore volume was 0.0430 cm³/g, besides the average pore diameter was estimated as 15.228 nm, confirming the mesoporous nature of this nanocomposite. The observed pore size is beneficial for diffusion-based processes and is consistent with the expected meso-structure formed by interpenetrating networks of chitosan, GO sheets, and embedded calcite particles.

#### Thermal stability investigation

The thermal behavior of the HCM@CH@GO nanocomposite was investigated by TGA under nitrogen atmosphere, and the corresponding thermogram is presented in Fig. 6. The TGA curve shows weight loss via multi-step profiles, indicating the decomposition of various components of the nanocomposite at different temperature ranges. The first significant weight loss (14.4%) was generated at 200 °C due evaporation of adsorbed water and volatile components, which are typically trapped into the porous hydrogel matrix and on the surface of GO sheets. This initial thermal event reflects the hydrophilic nature and high water retention capacity of the chitosan-based hydrogel. The second weight loss step, 8.9% centered at 390 °C, corresponds to degradation of chitosan polymer chains. This involves the cleavage of glycosidic linkages and decomposition of functional amine and hydroxyl groups, leading to the evolution of gaseous products as CO₂, NH₃, and small organic fragments [37]. Additionally, partial degradation of the oxygen-containing functional groups on GO may also contribute to this stage. The third step is the most significant degradation step that occurs at approximately 590 °C, with 23.3% loss, which is attributed to the breakdown of organic moieties. This stage may also involve the decomposition of carbonate groups from embedded calcite (CaCO₃) particles, releasing CO₂ and resulting in a noticeable mass loss. The residual mass at > 600 °C indicates the presence of thermally stable inorganic content, primarily calcium oxide (CaO), derived from the thermal decomposition of CaCO₃ [25]. This residue confirms the successful incorporation of calcite nanoparticles into the composite matrix and their contribution to the enhanced thermal stability.

### Adsorption of MoS_2_-QDs by HMC@CH@GO nanocomposite

The adsorption performance of HMC@CH@GO nanocomposite toward MoS₂-QDs removal (50 mg/L) under normal conditions (pH 7) was first evaluated and compared with the removal efficiency of the individual constituents: HMC (21.8%), CH (30.6%), GO (28.0%), and the binary composite HMC@CH (33.7%). As summarized in Table 3S, the ternary HMC@CH@GO nanocomposite exhibited the highest removal efficiency (41.4%). Such enhanced performance confirms that structural integration within the hybrid nanocomposite has markedly improved MoS₂-QDs adsorption, highlighting the superior removal capability and functional synergy of HMC@CH@GO.

#### Effect of pH on MoS_2_-QDs removal by HMC@CH@GO nanocomposite

Variation in solution pH has a significant influence on the stability and surface functionality of both MoS₂-QDs and the HMC@CH@GO nanocomposite. The colloidal stability of MoS₂-QDs generally increases with increasing pH by providing the optimal observed stability under neutral to mildly alkaline conditions. Under highly acidic conditions (pH ≤ 2), protonation of surface functional groups, oxidative transformation of Mo(IV) to Mo(VI) species (e.g., MoO₄²⁻ formation), and partial dissolution of MoS₂-QDs may occur. These processes can adversely affect the structural and colloidal stability of MoS₂-QDs and, consequently, influence the apparent removal efficiency.

**Fig. 7:**
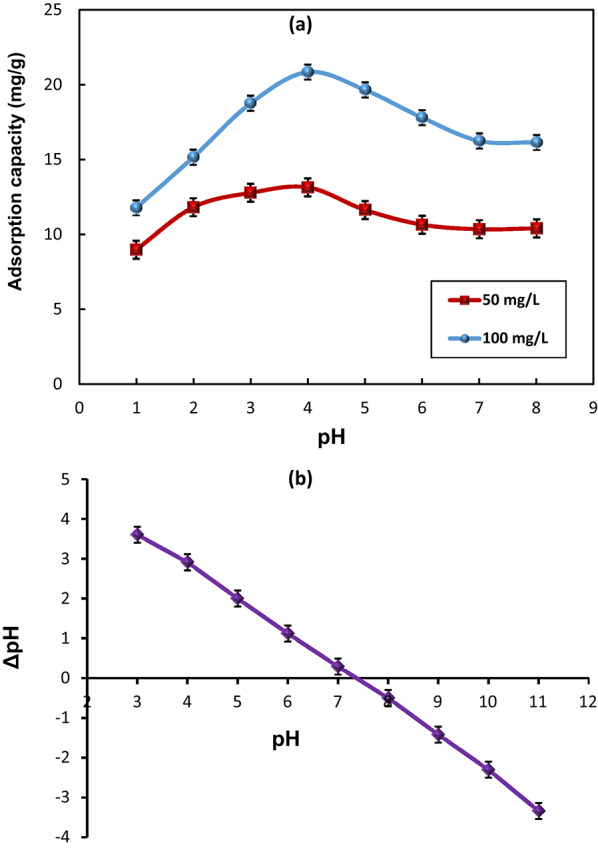
(**a**) Effect of pH on MoS_2_-QDs adsorption capacity, and (**b**) PZC of HMC@CH@GO nanocomposite

The effect of solution pH on the adsorption capacity of the HMC@CH@GO nanocomposite toward MoS₂ QDs was investigated over the pH range of 1–8, as shown in Fig. 7a. Two initial concentrations of MoS₂-QDs (50 and 100 mg/L) were examined to evaluate the consistency of the adsorption behavior. For both concentrations, the adsorptive capacity was increased progressively as the pH changed from 1 to 4, reaching a maximum at pH 4, followed by a gradual decline at higher pHs. Specifically, at 50 mg/L, the highest values of adsorption capacity attained was 13.15 mg/g, while at 100 mg/L, it reached approximately 21.0 mg/g. The consistent trend at both concentrations indicates that pH plays a critical role in modulating the surface charge on both the MoS₂-QDs and HMC@CH@GO species. The improved adsorption at acidic pH (≤ 4) could be attributed to increased protonation of –OH, –NH₂, and –COOH, located on the chitosan and GO components of the nanocomposite. Such protonated groups facilitate electrostatic attraction with the electron-rich MoS₂-QDs species, which are likely to exist as stable anionic colloids under such conditions [44]. In addition, π–π interactions between the GO nanosheets and MoS₂-QDs, as well as possible coordination interactions between QDs species and Ca²⁺ sites in the calcite phase, may enhance the overall adsorption process. Furthermore, the hierarchical mesoporous structure of the nanocomposite likely supports efficient diffusion and interaction of MoS₂-QDs with accessible binding sites. As the pH increases above 4, the adsorption capacity decreases, likely due to decreasing in surface protonation and increasing the electrostatic repulsion of negatively charged HMC@CH@GO surface with MoS₂-QDs. Additionally, at higher pH, the deprotonation of active sites may slightly hinder effective complexation and thus, weakening the adsorption affinity.

To express deeper insight via the pH impact on behavior of MoS₂-QDs, the point of zero charge (PZC) of HMC@CH@GO nanocomposite was determined, as illustrated in Fig. 7b. The ΔpH (pH_final_ – pH_initial_) was plotted against the initial pHs. The PZC is identified at the intersection where ΔpH = 0, which corresponds to a pH value of approximately 7.8. Accordingly, at lower pH values (< PZC), the composite surface is positively charged, favoring strong binding with negatively charged MoS₂-QDs via electrostatic attraction. However, at pH values > PZC, the surface turns to negative, leading to electrostatic repulsion and a consequent declining in adsorption efficiency. On the other hand, the higher adsorption observed at lower initial concentration (50 mg/L) compared to 100 mg/L also suggests the saturation of available binding sites at elevated concentrations, consistent with typical adsorption behavior.

#### Effect of HMC@CH@GO mass on MoS₂-QDs removal efficiency

**Fig. 8:**
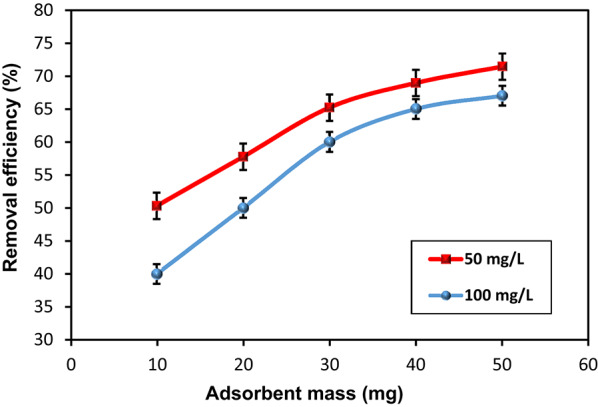
The effect of adsorbent mass on removal efficiency of MoS_2_-QDs by HMC@CH@GO

The influence of HMC@CH@GO mass on the 50 and 100 mg/L MoS₂-QDs uptake was illustrated in Fig. 8. The plotted data show that removal efficiency increased progressively with rising nanocomposite mass for both concentrations. At 50 mg/L, removal efficiency increased from approximately 50% to 72%, while at 100 mg/L, it improved from 40 to 67% as the HMC@CH@GO mass increased from 10 to 50 mg. The observed trend signify the increase in the number of binding centers and available surface area with higher HMC@CH@GO dosages. A greater quantity of nanocomposite provides more accessible functional groups (–OH, –COOH, –NH₂) capable of interacting with MoS₂-QDs through electrostatic attraction, hydrogen bonding, or surface complexation. However, the increase rate in removal efficiency becomes less pronounced beyond 30–40 mg HMC@CH@GO, especially for the 100 mg/L solution. This plateauing effect suggests the onset of adsorption site saturation, where nearly all accessible MoS₂-QDs have already been captured by the available active sites. In other words, excess nanocomposite does not lead to a proportionate increase in removal efficiency once equilibrium is approached [45]. Additionally, the removal efficiency is consistently higher at the lower initial concentration (50 mg/L), be due to a more favorable adsorbent-to-adsorbate ratio and reduced competition among for binding sites. Although higher dosages slightly increased the removal percentage, the adsorption capacity per unit mass decreased. Therefore, 30 mg is considered as the optimal dosage, providing a favorable balance between high removal efficiency and efficient nanocomposite utilization.

#### Temperature influence and thermodynamic analysis

**Fig. 9:**
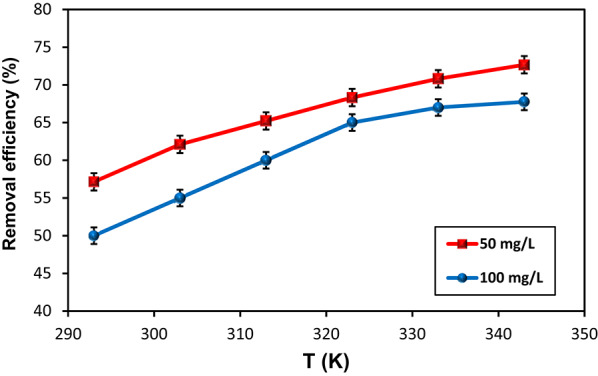
The effect of temperature on % removal efficiency of MoS_2_-QDs by HMC@CH@GO

The temperature influence on MoS₂-QDs uptake by the HMC@CH@GO nanocomposite was investigated across the temperature range from 298 to 343 K, using two initial concentrations: 50 and 100 mg/L. Figure 9shows that removal efficiency improved consistently with increasing temperature for both concentrations, indicating an endothermic adsorption process. At both concentrations, the % removal increased gradually as the temperature rose, suggesting enhanced interaction between MoS₂-QDs and active sites on the nanocomposite at elevated temperatures. Such behavior can be attributed to (i) increased molecular diffusion of MoS₂-QDs across the boundary layer due to decreased viscosity at higher temperatures, and (ii) activation of additional adsorption sites or structural rearrangements within the hydrogel network that enhance binding at elevated temperatures [46]. To further clarify the characteristics of the adsorption process, (ΔG°), (ΔH°), (ΔS°) were calculated using the Van’t Hoff expressions (3) and (4).

3 $$\:{\Delta\:}\mathrm{G}^\circ\:=-\mathrm{R}\mathrm{T}\mathrm{ln}\mathrm{K}\:\:\:\:\:\:\:\:\:\:\:\:\:\:\:\:\:\:\:\:\:\:\:\:\:\:\:\:\:$$

4 $$\:{\Delta\:}\mathrm{G}^\circ\:={\Delta\:}\mathrm{H}^\circ\:-\mathrm{T}{\Delta\:}\mathrm{S}^\circ\:\:\:\:\:\:\:\:\:\:\:\:\:\:\:\:\:\:\:\:\:\:\:\:$$

Where, *R* = 8.314 J/ mol.K, T = temperature (K) and K = equilibrium constant.

The thermodynamic parameters were characterized from the slope and intercept of the linear plot are summarized in Fig. 2S; Table 1. The equilibrium constant (K = q_e_/C_e_) used for the thermodynamic calculations was derived from the adsorption distribution coefficient. For both concentrations, more negative ΔG° values were realized with increasing temperature confirming the spontaneous nature with thermodynamically more favorable at higher temperatures. At 100 mg/L, ΔG° shifted from + 1.69 kJ/mol at 298 K to − 0.14 kJ/mol at 343 K, while at 50 mg/L it changed from + 0.99 kJ/mol to − 0.81 kJ/mol across the same temperature range. The positive ΔH° values (13.12 kJ/mol at 100 mg/Land 11.43 kJ/mol at 50 mg/L) confirm the endothermic nature of the adsorption process, implying that heat absorption enhances MoS₂-QDs binding. This is likely due to greater mobility of MoS₂-QDs and possibly increased swelling of the hydrogel matrix, exposing more active binding sites. However, the relatively low ΔH° values suggest that the process is primarily governed by physical interactions with possible contributions from surface complexation. The positive ΔS° values (39.32 J/mol·K at 100 mg/L and 35.90 J/mol·K at 50 mg/L) show an increase in randomness. This entropy gain suggests that the interaction may involve desolvation of water molecules and reorganization of the adsorbent surface upon MoS₂-QDs binding [20]. These thermodynamic findings support spontaneous process, favorable, heat-driven, and accompanied by increased disorder, aligning with the observed enhancement in removal efficiency at higher temperatures.

**Table 1:** Standard thermodynamic parameters of MoS_2_-QDs adsorption by HMC@CH@GO nanocomposite

MoS₂-QDs concentration	T (K)	K_D_	Adsorption thermodynamic parameters			*R* ^2^
			Ln Δ G° (kJmol^-1^)	Δ H° (kJmol^-1^)	ΔS° (Jmol^-1^K^-1^)	
100 mg/L	298	-0.69	1688.5		39.32	0.964
	303	-0.49	1240.6			
	313	-0.29	748.63			
	323	-0.07	199.01	13.12		
	333	0.02	-41.63			
	343	0.05	-140.19			
50 mg/L	298	-0.41	987.71		35.90	0.993
	303	-0.20	500.93			
	313	-0.06	167.95	11.43		
	323	0.08	-202.77			
	333	0.19	-534.07			
	343	0.28	-812.27			

#### Effect of contact time on MoS₂-QDs removal efficiency by HMC@CH@GO and kinetic models

**Fig. 10:**
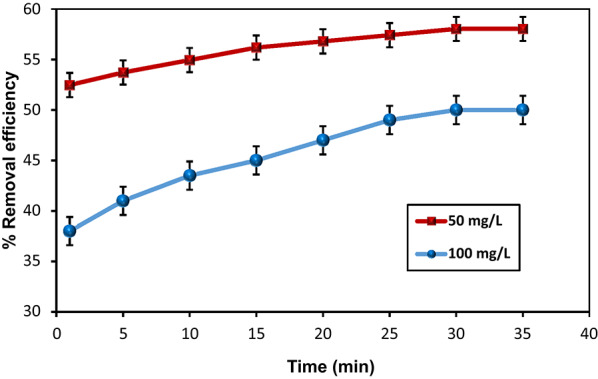
Effect of shaking-contact time on % removal efficiency of MoS_2_-QDs by HMC@CH@GO

The adsorption time contribution on the removal efficiency of MoS₂-QDs by HMC@CH@GO nanocomposite was investigated using 50 and 100 mg/L. As presented in Fig. 10, the removal efficiency increased rapidly during the first 20 min and followed by a slow process to approach toward equilibrium, at approximately 30 min by both concentrations. This behavior is characteristic of typical adsorption kinetics, where the abundant availability of active surface sites on HMC@CH@GO drives the initial rapid uptake. As the sites become progressively occupied, the adsorption rate slows down due to increased resistance to mass transfer and reduced concentration gradient.

The mechanism and rate-controlling steps were further elucidated by fixing the data to four kinetic models: pseudo-first order (PFO), pseudo-second order (PSO), intraparticle diffusion, and Elovich models as compiled in Table 2. The linear graphical representations of these models are shown in Fig. 3Sa-d. Despite the PFO model yielded reasonably good correlation coefficients (R² = 0.919 and 0.983for 100 and 50 mg/L), the calculated equilibrium adsorption capacities (qe_(calc)_ = 7.74 and 1.69 mg/g for 100 and 50 mg/L, respectively) deviated significantly from the experimental values (qe_(exp)_ = 25.00 and 14.51 mg/g). This suggests that the PFO model does not accurately represent the adsorption mechanism. However, The PSO model provided an excellent fit for both concentrations, with high R² values (0.997 for 100 mg/L and 0.999 for 50 mg/L), and the q_e(calc)_ values (25.71 and 14.64 mg/g) closely matched the experimental measurements. These results confirm that the binding of MoS₂-QDs onto the HMC@CH@GO nanocomposite involves valence forces through electron transfer or sharing between functional groups on the nanocomposite surface and MoS₂-QDs, indicating the chemisorption contribution [47]. The intraparticle diffusion model also showed strong correlation (R² = 0.990 for 100 mg/L and 0.988 for 50 mg/L), suggesting that diffusion into porous structure of the nanocomposite played a critical role in the overall adsorption process. The deviation of the plots from the origin suggests that intraparticle diffusion is involved but not the primary rate-controlling mechanism; instead, it occurs in combination with boundary layer diffusion [48]. The Elovich model yielded moderate fits (R² = 0.925 and 0.932), consistent with a heterogeneous surface adsorption mechanism. The high values of the Elovich constant α, especially for 50 mg/L (α = 1.04 × 10¹³ mg/g. min), suggest rapid adsorption, and followed by a deceleration as the surface becomes saturated. Overall, the analysis of the kinetic and thermodynamic data indicates that the adsorption of MoS₂-QDs onto the HMC@CH@GO nanocomposite was not governed by a single mechanism, but rather involves a combination of strong physical binding, weak chemical interactions, and diffusion phenomena.

**Table 2:** Kinetic models of MoS_2_-QDs adsorption by HMC@CH@GO nanocomposite

Kinetic model		Kinetic parameters	100 mg/L	50 mg/L
Pseudo-first order (PFO) $\:\mathbf{ln}\left({\boldsymbol{q}}_{\boldsymbol{e}}-{\boldsymbol{q}}_{\boldsymbol{t}}\right)=\boldsymbol{l}\boldsymbol{n}{\boldsymbol{q}}_{\boldsymbol{e}}-{\boldsymbol{k}}_{1}\boldsymbol{t}$		q_e(exp)_ (mg g^-1^), experimental adsorbed amount	25.00	14.51
		q_e(calc)_ (mg g^-1^), calculated adsorbed amount at equilibrium	7.74	1.69
		k_1_ (min^-1^), PFO rate constant	0.094	0.089
		R^2^	0.919	0.983
Pseudo-second order (PSO) $\:\frac{\boldsymbol{t}}{{\boldsymbol{q}}_{\boldsymbol{t}}}=\frac{1}{{\boldsymbol{k}}_{2}{\mathbf{q}}_{\mathbf{e}}^{2}}+\frac{\mathbf{t}}{{\mathbf{q}}_{\mathbf{e}}}$		q_e(exp)_ (mg g^-1^), experimental adsorbed amount	25.00	14.51
		q_e(calc)_ (mg g^-1^), calculated adsorbed amount at equilibrium	25.71	14.64
		k_2_ (g mg^-1^ min^-1^), PSO rate constant	0.009	0.127
		R^2^	0.997	0.999
Intraparticlediffusion $\:{\boldsymbol{q}}_{\boldsymbol{t}}={\boldsymbol{k}}_{\boldsymbol{i}\boldsymbol{d}}{\boldsymbol{t}}^{1/2}+\boldsymbol{C}$		K_id_ (mg. g^-1^ min^-1/2^), intraparticle diffusion rate constant	1.302	0.304
		C (mg g^-1^), thickness of the boundary layer	17.65	12.80
		R^2^	0.990	0.988
Elovich $\:{\boldsymbol{q}}_{\boldsymbol{t}}=\frac{1}{\boldsymbol{\upbeta\:}}\mathbf{ln}\left(\boldsymbol{\upalpha\:}\boldsymbol{\upbeta\:}\right)+\frac{1}{\boldsymbol{\upbeta\:}}\mathbf{ln}\mathbf{t}$		α (mg g^-1^ min^-1^), surface coverage for the initial rate of adsorption	4.93 × 10^4^	1.04 × 10^13^
		β (mg g^-1^ ), the activation energy of chemisorption	0.559	2.382
		R^2^	0.925	0.932

#### Effect of initial MoS₂-QDs concentration and isotherm modeling

**Fig. 11:**
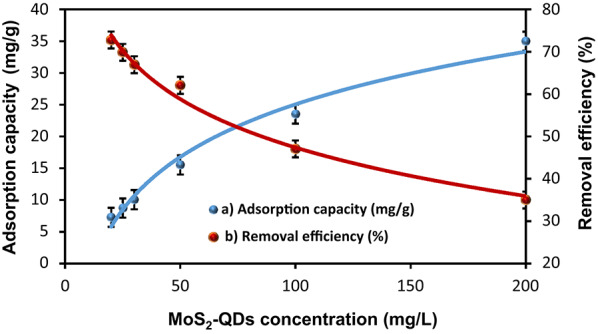
The effect of MoS2-QDs concentration on (**a**) adsorption capacity, and (**b**) %removal efficiency

The MoS₂-QDs concentration influence on the performance by HMC@CH@GO nanocomposite is depicted in Fig. 11a and b, which show trends in adsorption capacity and removal efficiency, respectively. The adsorption capacity increased with rising initial MoS₂-QDs concentration, indicating that high concentration enhanced the driving force for mass transfer from the solution to the solid surface. As more MoS₂-QDs are introduced into the system, a greater number of interactions occur between the MoS₂-QDs and surface free functional groups, resulting in greater uptake per unit mass of HMC@CH@GO. This trend is consistent with adsorption theory, where increasing the concentration gradient promotes faster and more extensive adsorption, especially in high surface area materials with accessible mesoporosity as HMC@CH@GO nanocomposite [49]. In contrast, the removal efficiency exhibited a decreasing trend as the initial MoS₂-QDs concentration increased. At lower concentrations, the number of free binding centers on the HMC@CH@GO surface significantly exceeds the quantity of MoS₂-QDs in solution, leading to high removal efficiency. However, as the concentration increases, the active sites become progressively saturated, and a portion of MoS₂-QDs remains unadsorbed in the solution.

The adsorption mechanism and nature of interactions between MoS₂-QDs and the HMC@CH@GO nanocomposite were further declared by fitting the equilibrium data to four classical patterns; Langmuir, Freundlich, Dubinin–Radushkevich (D–R), and Temkin. The model parameters and correlation coefficients (R²) are presented in Table 3, while their linear plots are shown in Fig. 4Sa–d. The Langmuir adsorption model predicts that adsorption occurs as a single molecular layer on a uniform surface containing a limited number of equivalent binding sites, and no lateral interactions between adsorbed species [50]. The high correlation coefficient (R² = 0.984) indicates a strong agreement with Langmuir model, suggesting that adsorption occurs via homogeneous surface of specific sites. The q_max_ was determined as 33.3 mg/g, indicating that the nanocomposite possesses a relatively high uptake potential for MoS₂-QDs. The Langmuir constant (b) was found 0.05 L/mg, reflecting a moderate chemical binding affinity between the MoS₂-QDs and HMC@CH@GO. Furthermore, the dimensionless separation factor, ranged between 0.51 and 0.09, which indicates the adsorption favorability. The Freundlich model is an empirical isotherm that describes adsorption on heterogeneous surfaces with the possibility of multilayer formation [50]. It yielded the highest correlation coefficient (R² = 0.992) among all models, indicating that the adsorption process is better represented by surface heterogeneity and non-uniform energy distribution. The Freundlich constant (*n* = 2.02) falls in a favorable condition (1 < *n* < 10), suggesting that the nanocomposite provides a good adsorption intensity and capacity. The K value (3.26 L/mg) also confirms the strong affinity of HMC@CH@GO nanocomposite toward MoS₂-QDs through physical interactions. The D–R model is used to investigate the nature of adsorption, whether it mainly proceeds physically or chemically, based on the mean free energy (E_s_) of adsorption. This model yielded a relatively low correlation coefficient(R² = 0.812), a theoretical adsorption capacity (q_s_=25.04 mg/g) and an energy value (E_s_=0.627 kJ/mol). Since E_s_< 8 kJ/mol, it is indicating the presence of physisorption [51]. Temkin model takes into account the interactions between adsorbate and adsorbent, assuming a linearly decrease in the adsorption heat with increasing coverage due to these interactions. The Temkin constant (b_T_ = 8.66 J/mol) of MoS₂-QDs adsorption by the HMC@CH@GO nanocomposite, indicates a moderate heat of adsorption, consistent with a physical adsorption mechanism [52]. Overall, the isotherm analysis demonstrated that both the Langmuir and Freundlich models provided an excellent match with the equilibrium data for MoS₂-QDs adsorption onto the HMC@CH@GO nanocomposite. These results indicate a strong contribution of physisorption, complemented by a chemisorption component that has been previously evidenced by the pseudo-second order pattern. These findings suggest that adsorption occurs on a heterogeneous surface with potential multilayer formation, while also involving specific homogeneous binding sites. Collectively, these observations confirm the high affinity of MoS₂-QDs toward the synthesized HMC@CH@GO nanocomposite, proceeding through multiple adsorption pathways.

**Table 3:** Isotherm parameters of different isotherm models for MoS_2_-QDs adsorption by HMC@CH@GO

Isotherm model	Isotherm parameters	
Langmuir $\:\frac{{\boldsymbol{C}}_{\boldsymbol{e}}}{{\boldsymbol{q}}_{\boldsymbol{e}}}=\frac{1}{{\boldsymbol{b}.\boldsymbol{q}}_{\boldsymbol{m}\boldsymbol{a}\boldsymbol{x}}}+\frac{{\boldsymbol{C}}_{\boldsymbol{e}}}{{\boldsymbol{q}}_{\boldsymbol{m}\boldsymbol{a}\boldsymbol{x}}}$ $\:{\boldsymbol{R}}_{\boldsymbol{L}}=1/(1+\boldsymbol{b}.{\boldsymbol{C}}_{\boldsymbol{o}})$	**q**_**max**_(mg g^− 1^), maximum adsorption capacity	33.3
	**b** (L mg^− 1^ ), Langmuir constants	0.05
	**R**_**L**_, Separation factor	0.51–0.09
	**R** ^**2**^	0.984
Freundlich $\:\boldsymbol{l}\boldsymbol{n}{\boldsymbol{q}}_{\boldsymbol{e}}=\boldsymbol{l}\boldsymbol{n}{\boldsymbol{K}}_{\boldsymbol{F}}+\frac{1}{\boldsymbol{n}}\boldsymbol{l}\boldsymbol{n}{\boldsymbol{C}}_{\boldsymbol{e}}$	**n**, intensity of adsorbent	2.02
	**K**_**F**_ (L mg^− 1^ ), Freundlich constant	3.26
	**R** ^**2**^	0.992
Dubinin-Radushkevich $\:\boldsymbol{l}\boldsymbol{n}\:{\boldsymbol{q}}_{\boldsymbol{e}}=\boldsymbol{l}\boldsymbol{n}\:{\boldsymbol{q}}_{\boldsymbol{s}}-\:\left({\boldsymbol{K}}_{\boldsymbol{a}\boldsymbol{d}}{\mathbf{\varepsilon}}^{2}\right)$ $\:\mathbf{\varepsilon}=\boldsymbol{R}\boldsymbol{T}\boldsymbol{l}\boldsymbol{n}(1+\frac{1}{{\boldsymbol{C}}_{\boldsymbol{e}}}\:)$ $\:{\boldsymbol{E}}_{\boldsymbol{s}}=\frac{1}{\sqrt{{2\boldsymbol{K}}_{\boldsymbol{a}\boldsymbol{d}}}}$	**q**_**s**_(mg g^− 1^), maximum adsorption capacity	25.04
	**K**_**ad**_ (mol^2^ kj^− 2^), D-R constant	1.27
	**E**_**s**_(kJ mol^− 1^), energy of adsorption	0.627
	**R** ^**2**^	0.812
Temkin $\:{\boldsymbol{q}}_{\boldsymbol{e}}=\frac{\boldsymbol{R}\boldsymbol{T}}{{\boldsymbol{b}}_{\boldsymbol{T}}}\boldsymbol{l}\boldsymbol{n}{\boldsymbol{a}}_{\boldsymbol{T}}+\frac{\boldsymbol{R}\boldsymbol{T}}{{\boldsymbol{b}}_{\boldsymbol{T}}}\boldsymbol{l}\boldsymbol{n}{\boldsymbol{C}}_{\boldsymbol{e}}$ $\:{\boldsymbol{q}}_{\boldsymbol{e}}=\boldsymbol{B}\:\boldsymbol{l}\boldsymbol{n}{\boldsymbol{a}}_{\boldsymbol{T}}+\boldsymbol{B}\:\boldsymbol{l}\boldsymbol{n}{\boldsymbol{C}}_{\boldsymbol{e}}$ $\:\boldsymbol{B}=\frac{\boldsymbol{R}\boldsymbol{T}}{{\boldsymbol{b}}_{\boldsymbol{T}}}$	**a**_**T**_ (L g^− 1^), Temkin isotherm equilibrium binding constant	0.354
	**b**_**T**_( mg L^− 1^), Temkin isotherm constant	0.286
	**B** (J mol^− 1^), the adsorption heat	8.66
	**R** ^**2**^	0.980

#### Reusability of HMC@CH@GO nanocomposite

**Fig. 12:**
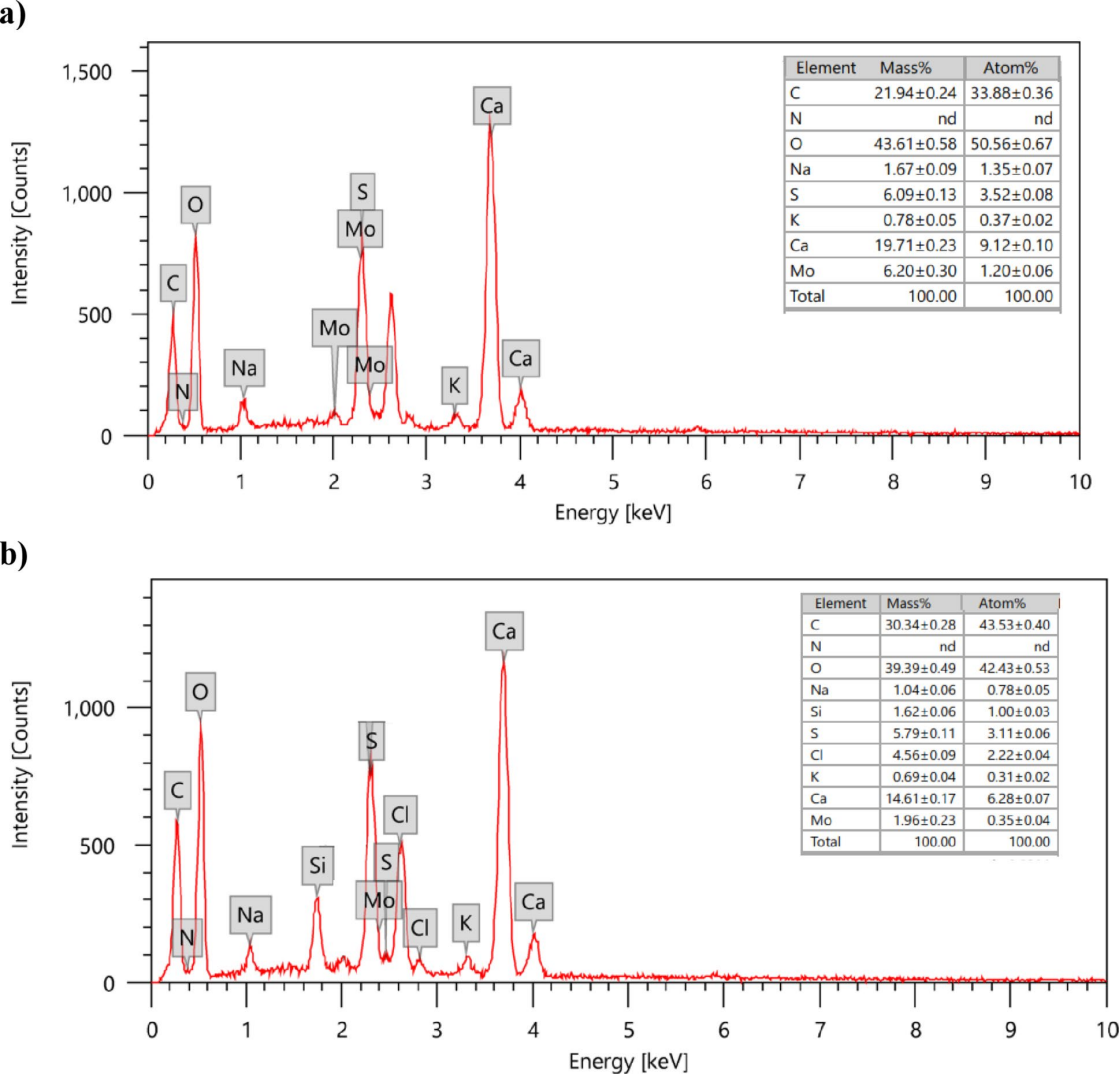
EDX spectra of (**a**) HMC@CH@GO loaded with MoS₂ QDs before regeneration and (**b**) regenerated HMC@CH@GO nanocomposite

The sustainability and long-term practical applicability of an adsorbent is largely depending on its regeneration and reusability over multiple cycles. The MoS₂-QDs desorption and regeneration of the HMC@CH@GO nanocomposite were performed using successive treatments with diluted NaOH and HCl solutions under controlled conditions. The alkaline treatment facilitated the desorption of adsorbed MoS₂-QDs from the composite surface, while subsequent acid washing restored the surface functionality of the nanocomposite. The regenerated material was thoroughly rinsed several times with distilled water to remove any residual eluent and then dried prior to reuse. The results confirmed the effective desorption of MoS₂-QDs and successful regeneration of the nanocomposite. As shown in Fig. 12a, the EDX spectrum of HMC@CH@GO after adsorption of MoS₂-QDs reveals the presence of Mo with a mass percentage at 6.2%, confirming the effective uptake and high removal efficiency of the nanocomposite toward MoS₂-QDs. In contrast, the EDX spectrum of regenerated HMC@CH@GO (Fig. 12b) demonstrates a significant reduction in Mo content to 1.9% (mass), indicating successful desorption during the regeneration process. Moreover, the relative elemental composition of the nanocomposite matrix exhibited slight change after regeneration, confirming the structural stability and integrity of the HMC@CH@GO. The successful adsorption-desorption of MoS₂-QDs and regeneration processes were further confirmed by FT-IR spectra of HMC@CH@GO before and after regeneration as shown in Fig. 5S.

**Fig. 13:**
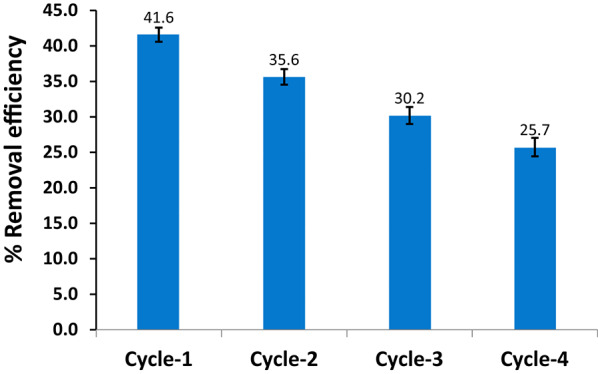
Reusability of HMC@CH@GO nanocomposite for removal of MoS_2_-QDs

The reusability of the HMC@CH@GO nanocomposite was evaluated through four consecutive adsorption–desorption cycles for removing 100 mg/L MoS₂-QDs. The results, illustrated in Fig. 13, revealed a gradual decrease in adsorption performance over successive cycles. In the first cycle, the HMC@CH@GO nanocomposite exhibited a removal efficiency of 41.6%, which progressively declined by approximately 15% with each subsequent cycle, reaching to 25.7% by the fourth cycle. This decline can be attributed to (i) partial loss or blockage of active sites on the composite surface due to incomplete desorption of MoS₂-QDs, and/or (ii) possible aggregation or surface fouling, which reduces accessible surface area and pore availability [31]. Despite the observed performance drop, the nanocomposite retained a moderate level of adsorption efficiency after four cycles.

#### Application of HMC@CH@GO in MoS₂-QDs removal from polluted water samples

To assess the practical performance of the HMC@CH@GO nanocomposite in wastewater treatment, its adsorption capability was evaluated in three different types of water matrices. The real water samples (tap water, seawater, and industrial wastewater) were used as collected without pH adjustment, and their initial pH values were measured to be at pH 6.5–7.5. The samples were not chemically modified before the adsorption experiments to better simulate realistic environmental conditions. The removal efficiency was assessed over three consecutive treatment cycles to evaluate performance stability under realistic conditions. The results are summarized in Table 4.In the first treatment cycle, removal efficiencies were 57.5% (tap water), 50.0% (seawater), and 43.5% (industrial wastewater). With repeated treatment runs, the efficiency improved significantly, reaching 84.0%, 73.0%, and 64.0% in tap water, seawater, and industrial wastewater, respectively, after the third cycle.

**Table 4:** Application of HMC@CH@GO inr removal of MoS_2_-QDs from real water samples

Water sample		Tap water	Sea water	Wastewater
% Removal	1st run	57.5	50.0	43.5
	2nd run	73.7	65.0	59.0
	3rd run	84.0	73.0	64.0

The lower removal efficiency observed in seawater and industrial wastewater compared to tap water can be attributed to matrix effects. Seawater is characterized by high ionic strength and elevated concentrations of competing ions such as Na⁺, Mg²⁺, Ca²⁺, Cl⁻, and SO₄²⁻. These ions may induce electrostatic screening, compress the electrical double layer, and compete with MoS₂-QDs for active adsorption sites, thereby weakening the electrostatic interactions between the nanocomposite surface and the target QDs. Similarly, industrial wastewater contains a complex mixture of dissolved organic matter (DOC), suspended solids, and inorganic contaminants. Organic molecules may be adsorbed by the nanocomposite surface, partially blocking active sites or forming a fouling layer that limits access of MoS₂-QDs to binding centers. In addition, multivalent ions present in wastewater may compete for coordination with surface functional groups (–OH, –COOH, –NH₂), to further declining the adsorption efficiency [33]. Despite these matrix interferences, the HMC@CH@GO nanocomposite maintained substantial removal performance across all water types, demonstrating its robustness and adaptability under environmentally relevant conditions.

## Conclusion

In this work, an innovative hierarchical mesoporous calcite–embedded chitosan hydrogel reinforced with graphene oxide (HMC@CH@GO) was successfully assembled and investigated in adsorptive removal of MoS₂-QDs from aqueous media. Structural analyses (FT-IR, XRD, SEM, TEM, EDX, BET, and TGA) confirmed the formation of a thermally stable, mesoporous hybrid framework with moderate area of 11.30 m²/g, pore diameter of 15.23 nm, and homogeneous dispersion of calcite nanoparticles within the hydrogel-GO matrix. Adsorption performance was strongly influenced by pH, adsorbent dosage, temperature, and initial MoS₂-QDs concentration, achieving a maximum capacity at pH 4, of 13.15 mg/g for 50 mg/L and 21.0 mg/g for 100 mg/L. Thermodynamic analysis revealed positive ΔH° values (13.12 kJ/mol and 11.43 kJ/mol for 100 and 50 mg/L, respectively) and increasingly negative ΔG° with temperature, confirming a spontaneous and endothermic process. Kinetic modeling showed an excellent fit to PSO, while intraparticle diffusion and boundary layer effects contributed to the overall rate. Isotherm analysis showed strong agreement with both Freundlich and Langmuir models, suggesting a heterogeneous surface with possible multilayer adsorption alongside specific homogeneous binding sites, with q_max_ = 33.3 mg/g. D–R analysis (Es = 0.627 kJ/mol) and Temkin constant (b_T_ = 8.66 J/mol) indicated that physisorption plays a critical role. The material retained ~ 25.7% removal efficiency after four reuse cycles and was effective in real-water applications, achieving high removal efficiencies across tap water, simulated seawater and wastewater. Overall, the novelty of this study is underscored by MoS₂-QDs remediation using an innovative HMC@CH@GO nanocomposite as a robust, multifunctional, and recyclable adsorbent. As summarized in Table 5, only a limited number of previous studies have specifically addressed the removal of related nano-pollutants.

**Table 5:** Comparative removal of nano-pollutants from water: previously reported studies and the present work

Nano-pollutant	Adsorbent	Optimum conditions/advantages	Ref.
Silver nanoparticles (Ag NPs)	Hierarchical mesoporous calcite (HMC)	pH 6–10; 480 min; q_max_ = 19 mg/g	[27]
Au/Ag-QDs	CoFe_2_O_4_ based magnetic covalent-organic framework	pH 5; 30 min; q_max_ = 121 mg/g; reusable	[13]
Ag-QDs	Magnetic CoFe2O4-biochar-polymeric nanocomposite	pH 5; Microwave heating time 25 s; q_max_= 243.9 mg/g	[15]
MoS_2_-QDs	HMC@CH@GO nanocomposite	pH 4; 30 min; q_max_ = 33.3 mg/g; reusable	Present study

## Supplementary Information

Below is the link to the electronic supplementary material.

Supplementary Material 1.

## Data Availability

All data generated or analyzed during this study are included in this published article and its supplementary information files.
