# Supplementary material for: Innovative adsorptive remediation of MoS₂-QDs from wastewater by decorated hierarchical mesoporous calcite@chitosan hydrogel@graphene oxide nanocomposite
Source: BMC Chem. 2026 May 7;20(1):99. doi: 10.1186/s13065-026-01811-3 (PMC13156882; doi:10.1186/s13065-026-01811-3)
Supplement: Supplementary file 1 — Supplementary Material 1. [file 13065_2026_1811_MOESM1_ESM.docx]

**Innovative adsorptive remediation of MoS₂-QDs from wastewater by decorated hierarchical mesoporous calcite@chitosan hydrogel@graphene oxide nanocomposite**

**Mohamed E. Mahmoud*, Mohamed F. Amira, Enass A.I. Saleh, Hany Abdel-Aal**

Faculty of Sciences, Chemistry Department, Alexandria University, Alexandria, Egypt.Email: memahmoud10@yahoo.com

Table 1S.Specifications of chemicals used in this study

| **Reagent** | **Chemical Formula** | **F.W (g/mol)** | **Assay** | **Company** |
| --- | --- | --- | --- | --- |
| Graphite fine powder particle size 150 mesh | C_x_ | 12.00x | ≥ 98.0% | Sigma–Aldrich Chemical Company, St Louis, USA |
| Chitosan  from shrimp shells | (C_6_H_11_O_4_N)_n_ |  | ≥75.0% |  |
| Ammonium molybdate tetrahydrate | (NH_4_)_6_Mo_7_O_24_ · 4H_2_O | 1235.86 | ≥ 99.9% |  |
| Ammonium sulfide | (NH₄)₂S | 68.14 | ≥ 20.0% |  |
| N-Methyl-2-pyrrolidone (NMP) | C_5_H_9_NO | 99.13 | ≥ 99.5% |  |
| Silica gel 60-120 mesh | SiO_2_ | 60.08 | ≥ 99.0% |  |
| Sodium nitrate | NaNO_3_ | 84.995 | ≥99.0% |  |
| Sulphuric acid | H₂SO₄ | 98.08 | ≥98.0% | Oxford Lab Fine Chem LLP, India |
| Phosphoric acid | H₃PO₄ | 97.99 | ≥85.0% |  |
| Hydrochloric acid | HCl | 36.46 | ≥37.0% |  |
| Sodium hydroxide | NaOH | 39.99 | ≥97.0 % |  |
| Ethanol | C_2_H_6_O | 46.07 | ≥ 99.5% |  |
| Acetic acid | C_2_H_4_O_2_ | 60.05 | ≥99.0% |  |
| Citric acid | C₆H₈O₇ | 192.12 | ≥99.5% |  |
| Potassium permanganate | KMnO_4_ | 158.03 | ≥99.0% |  |
| Hydrogen peroxide | H_2_O_2_ | 34.01 | ≥ 50.0% |  |
| Glutaraldehyde | C_5_H_8_O_2_ | 100.12 | ≥ 50.0% |  |
| Calcium chloride hexahydrate | CaCl_2_.6H_2_O | 219.08 | ≥98.0% |  |
| Sodium Carbonate | Na_2_CO_3_ | 105.99 | ≥99.5% |  |

**Table 2S.** Specifications of instrumental techniques used in this study

| **Instrumental technique** | **Model** | **Conditions** |
| --- | --- | --- |
| **FT-IR** | BRUKER VERTEX  70 Fourier Transform  infrared spectrophotometer | In the range 400–4500 cm−1 |
| **SEM** | Scanning electron  microscopic JSM6360LA, JEOL Ltd | A carbon tap was used as a substrate for SEM measurements using an ion sputtering coating device (JEOL-JFC-1100E) |
| **EDX** | Energy Dispersive X-ray  JSM-lT200, JEOL  Ltd | A carbon tap was used as a substrate for EDX measurements. Acceleration voltage 20.00 kV, WD 10.00 mm, Live time 30.00, high vacuum mode |
| **TEM** | Transmission electron  microscopy,model  JEOL JEM-2100F,  Japan | Acquiring the images at 80 to 200 kV |
| **XRD** | The X-ray diffraction by XRD Shimadzu lab X6100, Japan | The XRD generator worked at 40 kV,  30 mA, and λ = 1 Å utilizing target Cu-Kα with secondary monochromatic.2-Theta wa  s started at 10° and ended at 80°.The diffraction data was recorded with step of 0.02° and a time of 0.6 s at room temperature |
| **Thermal**  **Gravimetric Analysis (TGA)** | Perkin-Elmer TGA7 Thermobalance or by Linseis STAPT1000 using a temperature from 20 to 700 |  |
| **BET** | Brunauer–Emmett–  Teller by BELSORP-mini II, BEL J | The BET surface areas by nitrogen  adsorption–desorption isotherms were determined for 24 h |
| **UV/Vis**  **spectrophotometer** | Ultraviolet/visible  spectrophotometer by  V-530 JASCO | UV/ViS spectrophotometer in between the range of wavelength from 190 nm to 1100 nm was used in the absorption measurement |
| **pH-meter** | Adwa pH-meter | Standard buffers 4.01, 7.00 and 10.00 were utilized in the calibration of Adwa pH-meter which used in the measurements of solutions pH |
| **microwave oven** | KOG-1B5H, Korea 1400-W and 2.45 GHz |  |

**Table 3S.**Comparative study of MoS₂-QDs removal by individual constituents and the HMC@CH@GO nanocomposite adsorbent from a 50 mg L⁻¹ solution under neutral conditions (pH7).

| **Adsorbent** | **HMC** | **CH** | **GO** | **HMC@CH** | **HMC@CH@GO** |
| --- | --- | --- | --- | --- | --- |
| Removal efficiency (%) | 21.8 | 30.6 | 28.0 | 33.7 | **41.4** |


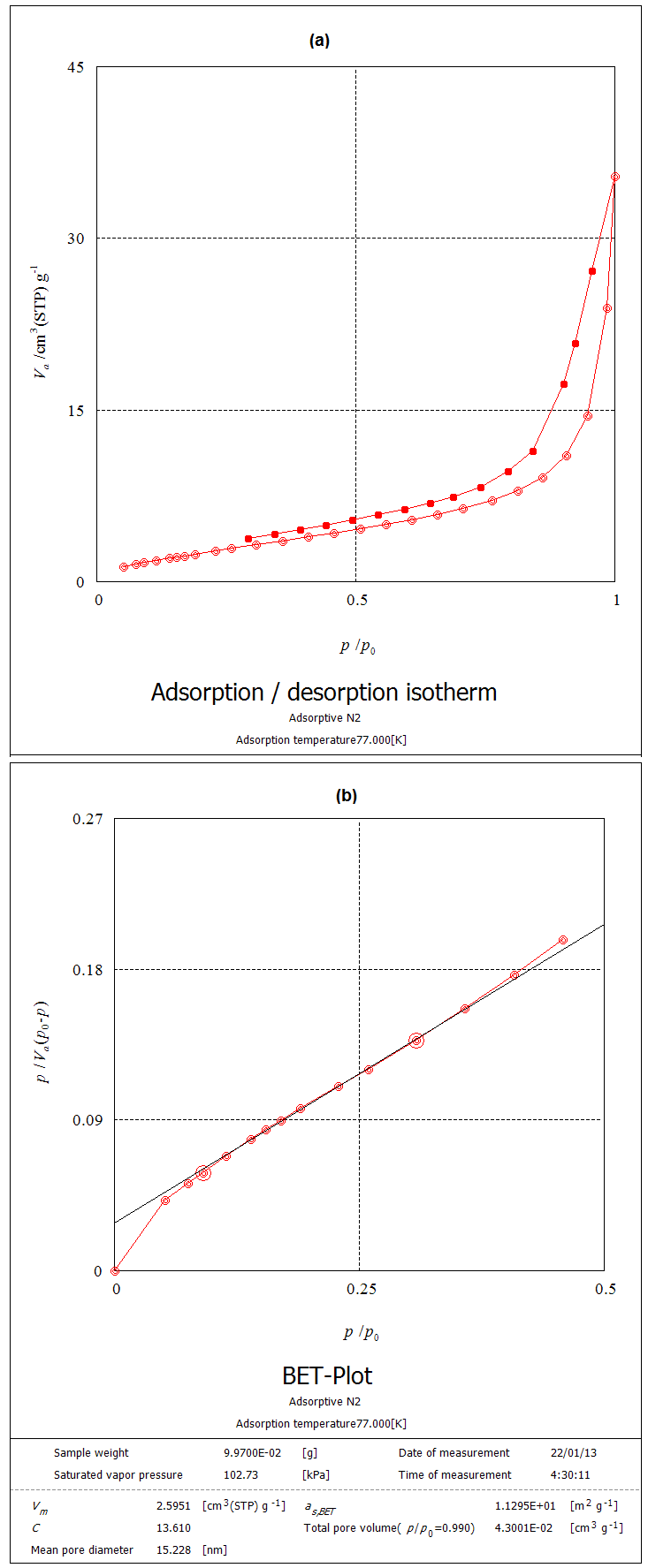


**Fig.1S.** (a) Adsorption/desorption BET isotherm and (b) BET linear plot, of HMC@CH@GO

**
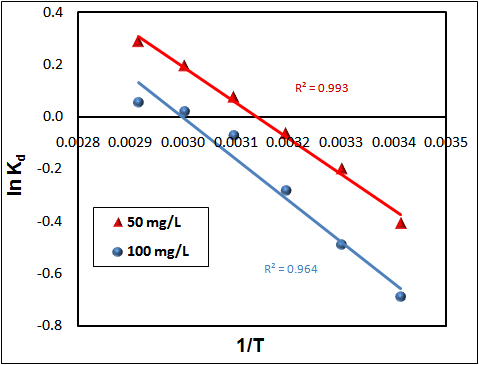
**

**Fig.2S.**Van’t Hoff plot for the adsorption of MoS_2_-QDs at pH 4 and 30 min shaking time

**
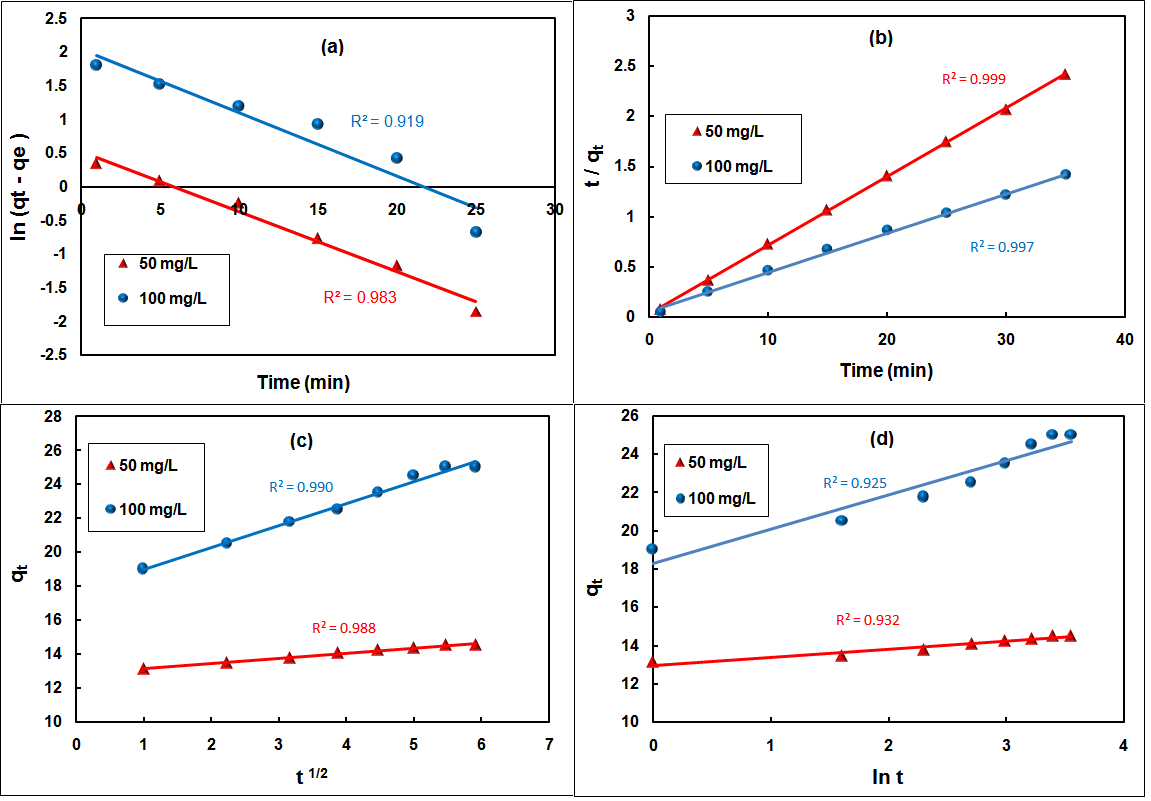
**

**Fig. 3S.** (a) Pseudo-first order, (b) Pseudo-second order, (c) Intraparticle diffusion and (d) Elovich models for kinetic studies of MoS_2_-QDs adsorption by HMC@CH@GO nanocomposite.


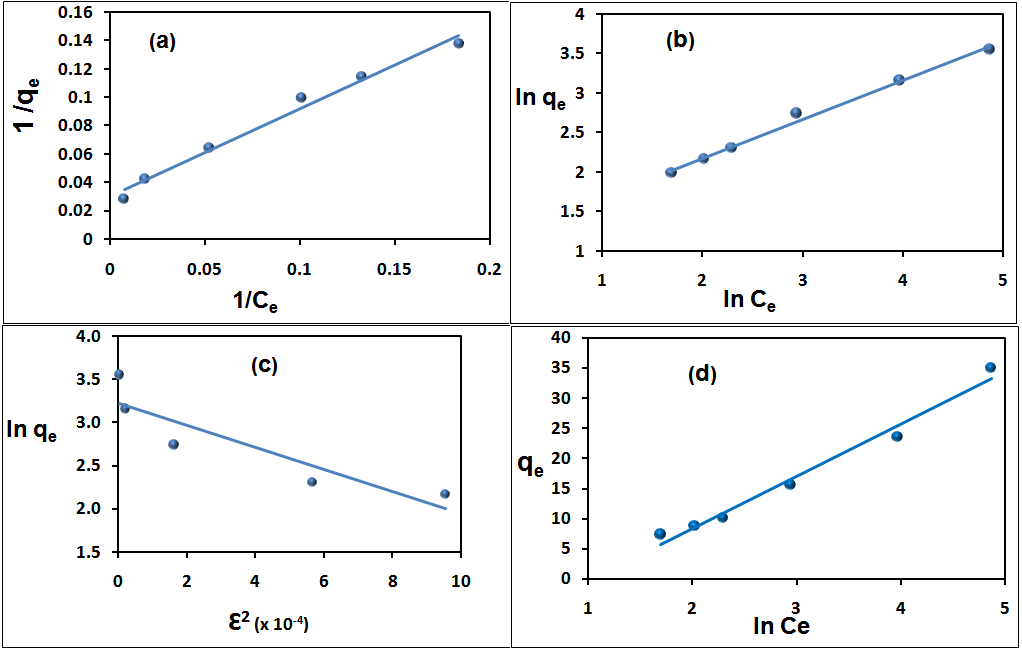
**Fig. 4S.**Adsorption isotherm models; (a) Langmuir, (b) Freundlich, (c) Dubinin-Radushkevich and (d) Temkin, for adsorption of MoS_2_-QDs on the adsorption capacity by HMC@CH@GO nanocomposite

| **a)**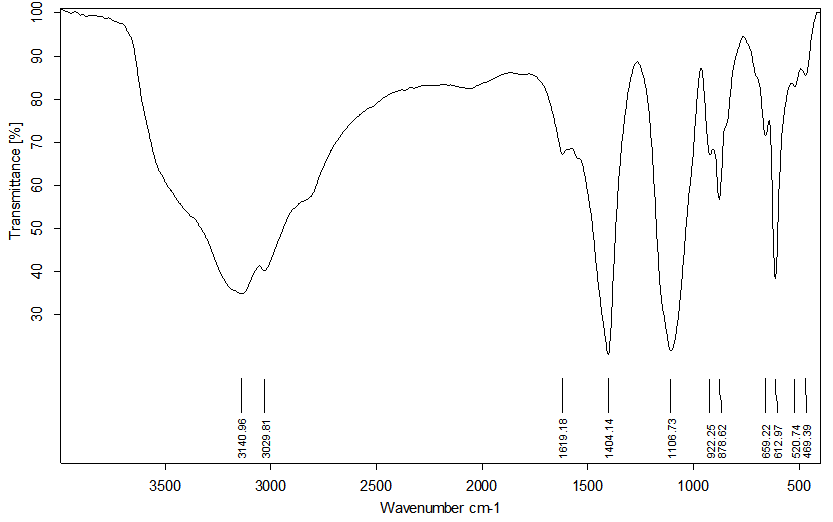 |
| --- |
| **b)**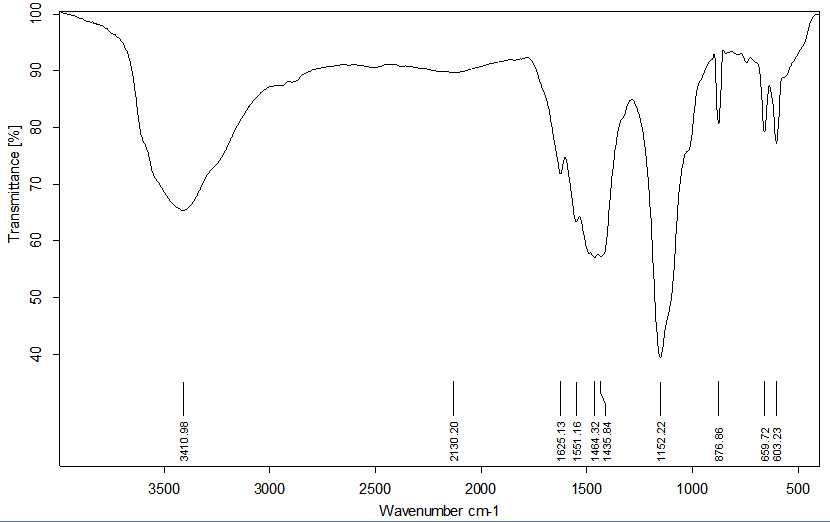 |
| **Fig. 5S.** FT-IR spectra of (a) HMC@CH@GO loaded with MoS₂ QDs before regeneration and (b) regenerated HMC@CH@GO nanocomposite. |
